# Carbon Mineralization in Fractured Mafic and Ultramafic Rocks: A Review

**DOI:** 10.1029/2023RG000815

**Published:** 2024-11-17

**Authors:** H. Nisbet, G. Buscarnera, J. W. Carey, M. A. Chen, E. Detournay, H. Huang, J. D. Hyman, P. K. Kang, Q. Kang, J. F. Labuz, W. Li, J. Matter, C. W. Neil, G. Srinivasan, M. R. Sweeney, V. R. Voller, W. Yang, Y. Yang, H. S. Viswanathan

**Affiliations:** ^1^ Earth and Environmental Sciences Division Los Alamos National Laboratory Los Alamos NM USA; ^2^ Civil and Environmental Engineering Northwestern University Evanston IL USA; ^3^ Department of Earth & Environmental Sciences University of Minnesota Minneapolis MN USA; ^4^ Department of Civil, Environmental, and Geo‐ Engineering University of Minnesota Minneapolis MN USA; ^5^ School of Civil and Environmental Engineering Georgia Institute of Technology Atlanta GA USA; ^6^ School of Ocean and Earth Science University of Southampton Southampton UK; ^7^ X‐Computational Physics Division Los Alamos National Laboratory Los Alamos NM USA

**Keywords:** carbon mineralization, fractures, CO_2_ storage, geochemistry, geomechanics, sequestration

## Abstract

Mineral carbon storage in mafic and ultramafic rock masses has the potential to be an effective and permanent mechanism to reduce anthropogenic CO_2_. Several successful pilot‐scale projects have been carried out in basaltic rock (e.g., CarbFix, Wallula), demonstrating the potential for rapid CO_2_ sequestration. However, these tests have been limited to the injection of small quantities of CO_2_. Thus, the longevity and feasibility of long‐term, large‐scale mineralization operations to store the levels of CO_2_ needed to address the present climate crisis is unknown. Moreover, CO_2_ mineralization in ultramafic rocks, which tend to be more reactive but less permeable, has not yet been quantified. In these systems, fractures are expected to play a crucial role in the flow and reaction of CO_2_ within the rock mass and will influence the CO_2_ storage potential of the system. Therefore, consideration of fractures is imperative to the prediction of CO_2_ mineralization at a specific storage site. In this review, we highlight key takeaways, successes, and shortcomings of CO_2_ mineralization pilot tests that have been completed and are currently underway. Laboratory experiments, directed toward understanding the complex geochemical and geomechanical reactions that occur during CO_2_ mineralization in fractures, are also discussed. Experimental studies and their applicability to field sites are limited in time and scale. Many modeling techniques can be applied to bridge these limitations. We highlight current modeling advances and their potential applications for predicting CO_2_ mineralization in mafic and ultramafic rocks.

## Key Points

- Development of carbon mineralization operations in mafic and ultramafic rocks requires integrating field, laboratory, and modeling studies
- Fractures play an integral role in the mineralization of CO_2_ in mafic and ultramafic rocks and must be considered in future studies
- Coupled geomechanical and geochemical processes in fractured mafic and ultramafic rocks require further experimental and modeling studies

## 1 Introduction

**Figure 1:**
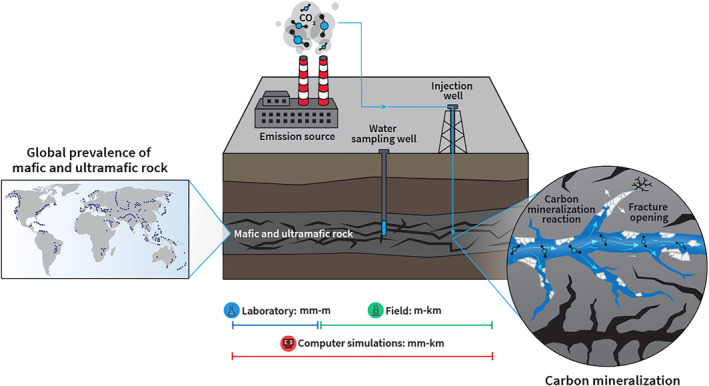
Carbon mineralization in mafic and ultramafic rocks is a promising strategy to permanently remove and store emitted CO_2_ into the subsurface due to the prevalence of mafic and ultramafic rocks worldwide, and the ability for these rocks to rapidly convert CO_2_ into minerals. This review highlights recent developments toward understanding the geochemical and geomechanical processes at the laboratory, field, and simulation scale.

A promising strategy to reduce anthropogenic CO_2_ is to permanently mineralize carbon in mafic and ultramafic geologic reservoirs. These rock masses are advantageous due to their prevalence in the earth's subsurface and their ability to rapidly store CO_2_ through carbon mineralization. Mafic and ultramafic rocks contain highly reactive silicate minerals abundant in metal cations (Mg^2+^, Ca^2+^, and Fe^2+^). When acidic CO_2_‐charged water reacts with these rock types, dissolution of the silicate minerals is promoted, releasing the cations into the pore fluid, where they can react with dissolved carbonate ions to precipitate carbonate minerals such as calcite (CaCO_3_), magnesite (MgCO_3_), siderite (FeCO_3_), and ankerite [Ca(Fe, Mg, Mn) (CO_3_)_2_], “locking” the carbon in the subsurface (Figure 1) (Snæbjörnsdóttir et al., 2020; Xiong, Wells, & Giammar, 2017; Xiong, Wells, Menefee, et al., 2017). Successful pilot‐scale mineral carbon storage projects in mafic rock, including CarbFix and CarbFix2 in Iceland (Clark et al., 2020; Pogge von Strandmann et al., 2019) and the Wallula basalt sequestration site in Washington, USA (S. K. White et al., 2020), have demonstrated rapid storage via mineralization on 2−3 year time scales. However, storing the levels of CO_2_ needed to address the present climate crisis requires a significant up‐scaling of these operations. An important consideration is how the CO_2_ is injected into the reservoir. The injection of CO_2_ that has been already dissolved in water, as done at the CarbFix sites, has the advantage of a more rapid conversion to mineral form, given that the CO_2_ has already achieved “solubility trapping” prior to injection (Snæbjörnsdóttir et al., 2020). However, the requirement for immense quantities of water cannot be forgotten and may not be favorable in other locations. This issue can be avoided by injecting CO_2_ as a supercritical fluid, as done at the Wallula basalt sequestration site. However, mineralization is expected to take longer, and requires favorable geologic formations that do not offer the possibility of leakage (Snæbjörnsdóttir et al., 2020). Therefore, determining the optimal injection conditions and having the ability to predict the impact of long‐term, large‐scale CO_2_ mineralization on geologic reservoirs is pertinent to up‐scaling CO_2_ mineralization operations.

Promising targets for subsurface mineralization are basalt (mafic) and peridotite (ultramafic). Basalt, in particular, volcanic lava flows and flood basalts which have already been tested at the pilot scale, has the advantage of possessing inter‐layer and fracture permeability that readily accommodates the injection of CO_2_ (Raza et al., 2022). However, a significant fraction of the total mass of basalt contained in the flow centers is impermeable (Aubele et al., 1988). Peridotite has the advantage of containing a greater concentration of divalent cations, making the rock more reactive, but it is also not as widespread and generally has lower permeability (Raza et al., 2022). Thus, for both basalt and peridotite, efficient mineralization of the rock mass requires a penetrating fracture network or the development of fractures that accommodate the flow and reaction of CO_2_‐bearing fluids. This involves optimizing fully coupled thermal, hydrological, mechanical, and chemical processes that can sustain flow for long‐term carbon mineralization. Complex feedbacks exist among fracture propagation, fluid flow, dissolution, precipitation, and fracture closure, including phenomena such as passivation of mineral surfaces that reduce reactive surface area (Béarat et al., 2006); carbonate precipitation that can clog pores and fractures (Jöns et al., 2017); subcritical fracture growth (Atkinson, 1984); and reaction‐driven cracking (Kelemen & Hirth, 2012). These questions require a coupled understanding of fluid flow and transport in fractured media and the chemical and mechanical processes that occur during carbon mineralization.

Mineral carbon storage is a rapidly developing field of research, which has fostered the publication of review papers providing a broad overview of storage potential (Aminu et al., 2017; Oelkers & Gislason, 2023; Power et al., 2013; Snæbjörnsdóttir et al., 2020) and geochemical processes, primarily as they relate to porous materials (DePaolo & Cole, 2013; L. Zhang et al., 2019; Y. Zhang et al., 2019). There is also extensive research on flow and fracture processes that apply to impermeable materials, including reviews on (a) the physical characteristics of fractures and fracture patterns (NRC, 1996), (b) hydromechanical‐thermomechanical processes in fractured rock (C. F. Tsang, 1991), and (c) flow and transport in fractured rock (Viswanathan et al., 2022; J. S. Y. Wang, 1991). To date, a review linking knowledge of fluid flow and transport, geochemistry, and the dynamic evolution of fractures during carbon mineralization in mafic and ultramafic rocks has not been published.

This review discusses recent developments in hydrological, geochemical, and geomechanics research domains highlighting new field, laboratory, and modeling studies that are relevant for carbon mineralization, combined with commentary on the implications for carbon mineralization in fractured mafic and ultramafic rocks. The current challenges that hinder our ability to accurately predict the outcome of long‐term carbon mineralization in these rock masses will also be discussed. The review is organized as follows:

Section 2. **Field Studies**. Carbon mineralization in mafic and ultramafic rocks has been recently investigated at the pilot scale. At CarbFix in Iceland, over 95% of the injected CO_2_ was mineralized in basalt after 2 years, while CarbFix2 mineralized >50% and 76% of the injected CO_2_ and hydrogen sulfide, respectively, in 4 to 9 months, and is currently still in operation (Clark et al., 2020; Matter et al., 2016). The Wallula Basalt Project in Washington State, USA mineralized 60% of CO_2_ in basalt after 2 years (S. K. White et al., 2020), while the injection of CO_2_ into peridotite‐rich Oman ophiolite is currently underway. Field observations and data derived from these tests provide invaluable information about the physicochemical processes occurring in the subsurface during and after CO_2_ injection, which can guide experimental designs and inform model parameters. In this review, we delve into the key findings from these field tests and highlight the variances in site characteristics including rock types, fracture networks, carbon mineralization products, fluid composition, and rates of mineralization. In addition, we outline the current knowledge gaps and challenges that inhibit the scale‐up of carbon mineralization to store the gigaton‐per‐year levels required to meet the needs of the climate crisis.

Section 3. **Experimental Studies**. Laboratory testing provides an opportunity to decipher the coupled flow, chemical and mechanical processes that occur during CO_2_ injection and subsequent carbon mineralization in a controlled environment. An abundance of laboratory studies, including flow‐through and batch‐reactor experiments, have been carried out to understand the geochemical reactions that facilitate the carbonation of mafic and ultramafic rocks, producing data such as mineral dissolution and precipitation kinetics, mineralogy of reaction products, hydrodynamic parameters (e.g., Damköhler and Peclet numbers), and surface characteristics. Fundamental research on the mechanical behavior of mafic and ultramafic rocks exists, which can be combined with studies on other materials (e.g., analog systems) that examine clogging and passivation of mineral surfaces, stress‐corrosion cracking, and reaction‐driven cracking, to understand fluid flow and reactive transport in fractured rock. We describe the advances in experimental studies on the geochemistry of carbon mineralization in analog and real geomaterials under relevant reservoir conditions, and fluid flow and reactions in fractures, as well as address the challenges of accurately representing field‐scale carbon mineralization processes in laboratory‐scale experiments.

Section 4. **Modeling and Simulation Studies**. Field observations and controlled laboratory experiments constrain the multi‐scale numerical models operating at the pore, single fracture, and fracture network scale that are used to predict the long‐term carbon mineralization of a reservoir. The coupled geochemical‐geomechanical processes that affect fracture creation and damage, fluid flow, and mineralization in current models do not include all the relevant processes. Numerous models exist that predict the evolution of fractures due to stress effects. Different models also exist to simulate flow through fractures. These models range in complexity from simple analytical models to mechanistic numerical models run on supercomputers. Few models couple flow and chemical reactions, while fully coupled studies of fracture, flow, and reaction are rare. For mafic and ultramafic rocks, answering key questions, such as whether stress‐corrosion cracking or reaction‐driven cracking will occur, requires a fully coupled approach. Models that are capable of describing fracture propagation rigorously with simplified assumptions for flow, transport, and reaction processes have been used for applications such as hydraulic fracturing. Other models that simulate flow, transport, and reactions once the fractures are created also exist and have been used for enhanced geothermal systems and nuclear waste disposal. We describe a combination of reduced complexity models, the latest generation of high‐performance computing physics‐based models, and statistically based uncertainty quantification (UQ) techniques that together can be used to better understand first‐order mechanisms to forecast carbon mineralization in reservoirs.

## 2 Field Studies of Carbon Mineralization in Mafic and Ultramafic Rock

### 2.1 Introduction

Over the past two decades, carbon mineralization in mafic and ultramafic rocks has developed from a conceptual idea to successful pilot‐scale operations globally (Clark et al., 2020; Kelemen, Matter, et al., 2020; Kelemen, McQueen, et al., 2020; Matter et al., 2016; McGrail et al., 2011). Motivated by the naturally reactive nature of mafic and ultramafic rocks, these exploratory field sites offer a critical first‐hand look at the feasibility and effectiveness of storing CO_2_ in these rock masses. Among mafic/ultramafic rocks, subsurface basalt (mafic) and peridotite (ultramafic) have been tested at various pilot injection sites, which we review in this section. CarbFix (Section 2.2), the inaugural CO_2_ mineralization project located in western Iceland's basalt, injected 230 tons of CO_2_ and a CO_2_‐H_2_S gas mixture dissolved in water (Matter et al., 2016), where an estimated ∼95% of the CO_2_ was mineralized after 2 years. The success of this operation resulted in the development of the CarbFix2 site, an up‐scaled commercial injection site that is showing promise. In Eastern Washington, USA, the Wallula Basalt Sequestration Project (Section 2.3) was carried out and was the first site to inject supercritical CO_2_ directly into flood basalts without first dissolving it in water. Here, after 2 years of injection, ∼60% of the CO_2_ was mineralized (S. K. White et al., 2020). More recently, the injection of CO_2_ dissolved in water into peridotites in Oman (Section 2.4) is being tested and monitored.

The success of carbon mineralization field studies demonstrates the viability of storing CO_2_ in mafic and ultramafic rocks. However, reaching the levels of stored CO_2_ needed to reduce atmospheric CO_2_ requires a significant up‐scaling of the operations and the introduction of new field sites. Key parameters that are currently poorly understood govern the feasibility of injection sites, including the existing and induced fracture network that provides a conduit for fluid and solute transport, the geochemical properties of the reservoir including the mineral reaction rates and reactive surface area, and water availability and use. Basalt inter‐layer zones tend to be highly porous and fractured, resulting in a “double‐edged sword” effect where, while these sites have high CO_2_ injectivity, they also have a greater potential for CO_2_ leakage. In the case of CarbFix, this issue was avoided by injecting CO_2_ already dissolved in water, reducing the risk of leakage through solubility‐trapping (Snæbjörnsdóttir et al., 2020). However, the high quantity of water used for injection may limit the scalability of the site to inject larger amounts of CO_2_. At Wallula, the injection of supercritical CO_2_ eliminates the need for large quantities of water, however, careful reservoir characterization and highly impermeable (unfractured) basalt flow centers capping the injection zone are needed to prevent CO_2_ migration (McGrail, Sullivan, et al., 2009). Furthermore, it is still not known how supercritical CO_2_, often considered inert to chemical reactions in the absence of water, will govern the distribution and capacity of CO_2_ mineralization within these rock masses during longer‐term injection (McGrail, Schaef, et al., 2009). Peridotites are more reactive rocks than basalt, and thus, have a higher potential to store CO_2_. However, these rocks tend to be relatively impermeable, and, thus, their effectiveness at mineralizing CO_2_ will likely be contingent on a connected network consisting of pre‐existing fractures, enhanced by hydraulic stimulation and aided by reaction‐driven fracturing and sub‐critical fracturing (discussed in Section 3.4). The total mass of CO_2_ that can be stored in mafic and ultramafic reservoirs is also governed by the physicochemical properties of the rock.

For both basalt and peridotite, the relative reactivity of minerals and the available reactive surface area are important factors that will influence the amount of CO_2_ mineralized at a given site. Furthermore, the precipitation of carbonates could lead to the passivation of reactive surfaces or the clogging of fluid pathways (Béarat et al., 2006; Jöns et al., 2017). Thus, analysis of coupled geochemical reactions, fluid flow, and the development of fracture networks in these systems is imperative. While it is impossible to visualize precisely what is occurring in the subsurface, we highlight key takeaways from the field sites discussed in the following sections and outline challenges (Section 2.5) that must be overcome to expand to large‐scale carbon mineralization operations.

### 2.2 The CarbFix Project

#### 2.2.1 Site Description

The CarbFix project was founded in 2007 by Reykjavik Energy, the University of Iceland, Columbia University, and CNRS Toulouse France. The aim of the CarbFix project was to demonstrate rapid CO_2_ mineralization in basaltic rocks, which was achieved on different scales by the CarbFix1 and CarbFix2 pilots from 2013 to 2020 (e.g., Aradóttir et al., 2012; Clark et al., 2020; Gislason et al., 2010; Matter et al., 2016; Pogge von Strandmann et al., 2019; Sigfusson et al., 2015; Snæbjörnsdóttir et al., 2017). The CarbFix1 pilot injection site, located approximately 2 km west of the Hellisheiði geothermal power plant, consists of a series of lava flows and glassy hyaloclastite formations (Alfredsson et al., 2013). The site is equipped with a 2,000 m deep injection well (HN02) and eight monitoring wells with depths ranging from 150 to 1,300 m. The injection operation focused on utilizing HN02 as the injection well and HN04, an inclined borehole, as the closest monitoring well. The distance between the two wells is ∼60 m at 400 m, 150 m at 650 m, and 360 m at 800 m depth, respectively (Alfredsson et al., 2013). The target CO_2_ storage formation was between 400 and 800 m depth, consisting of olivine tholeiitic basaltic lavas and hyaloclastites. The lateral and vertical intrinsic permeabilities were 300 and 1,700 × 10^−15^ m^2^, respectively (Aradóttir et al., 2012). A tracer test in combination with borehole logs revealed three distinct major flow paths or channels between wells HN02 and HN04, with the first flow path being located at 400 m, the second at 650 m, and the third at 850 m depth, respectively (Rezvani Khalilabad et al., 2008). The water level in the HN02 injection well was at ∼100 m depth, and the groundwater temperature and pH in the target storage formation ranged from 20 to 33°C and from 8.4 to 9.4, respectively (Rezvani Khalilabad et al., 2008).

The CarbFix2 injection site is located 1.5 km north of the Hellisheiði geothermal power plant and is utilizing pre‐existing multiple directionally drilled wells from the Hellisheiði geothermal field as injection and monitoring wells with total depths ranging from 2,204 to 2,606 m (Gunnarsson et al., 2018). The reservoir rocks consist of olivine tholeiitic basalt, with the top 1,000 m being dominantly hyaloclastites. The target storage reservoir is at depths greater than 1,300 m with an in‐situ temperature of >250°C (Gunnarsson et al., 2018). Fluid flow within the reservoir is controlled by multiple high‐permeability fractures and faults that intersect the injection and monitoring wells, as a result of the emplacement of intrusive rocks cross cutting the lava flows and hyaloclastites (Gunnarsson et al., 2018). In addition, fluid migration within the reservoir is influenced by the hydraulic gradient imposed by the far‐field injection and production wells (Ratouis et al., 2022). Tracer tests confirmed fast‐flowing pathways from the injection to the monitoring wells along NE‐SW trending faults and fractures (e.g., Gunnarsson et al., 2018).

#### 2.2.2 CO_2_ Injection and Post‐Injection Monitoring

CarbFix1 consisted of two injections, which were conducted in 2012. Phase 1 involved the injection of 175 tons of CO_2_ from January to March 2012, while Phase 2 involved the injection of 73 tons of CO_2_‐H_2_S gas mixture from June to August 2022, of which 55 tons were CO_2_. Due to the relatively shallow depth of the target storage reservoir, injection of buoyant supercritical CO_2_ into the fractured basalt reservoir was not possible. For this reason, a novel injection system of separately injecting CO_2_ and H_2_O at a ratio that facilitated complete solubility of CO_2_ into the water at the target depth was developed (Sigfusson et al., 2015). The water for the injection was sourced from the target storage reservoir. Injectivity at the target depth of 500–800 m was high enough for the injected water to flow down the injection wellbore by gravity alone. In the injection well, CO_2_ was released into the downflowing water through a sparger at a depth of 350 m, applying a CO_2_ injection pressure just above 25 bars. To assure complete dissolution of the CO_2_ before arriving in the target storage reservoir, the H_2_O:CO_2_ ratio was chosen to be greater than the CO_2_ solubility at the release depth. Furthermore, the water velocity in the injection well and thus the water injection rate was critical to counteract the buoyancy of the CO_2_ gas. Typical injection rates were ∼260 L/s for CO_2_ and ∼6,800 L/s for H_2_O during Phase 1 and between 38 and 190 L/s for CO_2_ and 1,578 and 7,881 L/s for H_2_O during Phase 2 (Matter et al., 2016).

The mineralization of the injected CO_2_ was monitored by co‐injecting a suite of chemical and isotopic tracers. The injected CO_2_ was spiked with radiocarbon (^14^C) to monitor its transport and reactivity in the reservoir. The ^14^C concentrations of the injected fluids were 40.0 Bq/L (^14^C:^12^C 2.16 × 10^−11^) during Phase 1 and 6 Bq/L (^14^C:^13^C 6.5 × 10^−12^) during Phase 2 (Matter et al., 2016). In addition, non‐reactive but volatile sulfur hexafluoride (SF_6_) and trifluoromethyl sulfur pentafluoride (SF_5_CF_3_) tracers were co‐injected with the CO_2_ to monitor injection plume migration and conservative mixing between injectate and reservoir fluid. Fluid samples for cation, anion, trace element, dissolved inorganic carbon, and tracer analyses were collected using an in‐situ downhole sampler in the injection well, and with a submersible pump from monitoring well HN04 during and after the dissolved CO_2_ injection.

CarbFix2 was designed to demonstrate the technical maturity and economic feasibility of CO_2_ and H_2_S capture from emission sources and permanent geologic storage via mineralization. Exhaust gas from the geothermal powerplant was dissolved in condensate in a scrubbing tower at 5–6 bar pressure (Gunnarsson et al., 2018). Subsequently, the CO_2_ and H_2_S‐charged condensate water was pressurized to 9 bar and transported to the CarbFix2 injection wells (Gunnarsson et al., 2018). The gas‐charged condensate water was injected to a depth of 750 m in separate stainless‐steel tubing in the injection wells at a rate of 30–60 L/s, where it was released into the downflowing geothermal brine from the powerplant (Clark et al., 2020). The inert tracer, 1‐naftalenesulfonic acid (1‐ns) was co‐injected with the gas‐charged condensate water to monitor injection plume migration and mixing (Clark et al., 2020). Steam and water phase samples were collected at the monitoring wells during the injection for geochemical analyses. By the end of 2017, 23,200 metric tons of CO_2_ and 11,800 metric tons of H_2_S had been injected. Injection at the CarbFix2 site continued after 2017 and by the end of 2023, over >97,000 tons of CO_2_ had been injected into the Hellisheiði geothermal reservoir (www.carbfix.com).

#### 2.2.3 Key Findings

**Figure 2:**
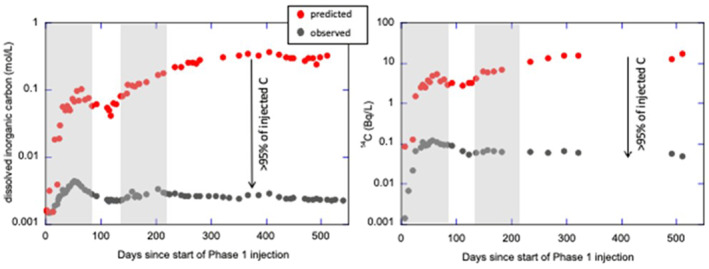
CarbFix1 mass balance calculations that indicate that >95% of dissolved CO_2_ was mineralized along the subsurface flow path from the injection to the monitoring well within two years. The figure depicts the predicted and observed dissolved inorganic carbon concentrations and isotope ratio of ^14^C in water at the CarbFix1 monitoring well. Predicted values are based on conservative mixing between the injectate and reservoir fluid using non‐reactive tracers such as SF_6_. The differences between predicted and observed values are consistent with the loss of almost all injected CO_2_ to form solid carbonate minerals along the flow path (modified from Matter et al. (2016)).

The injection of acidic CO_2_‐charged water results in the dissolution of the basalt host rocks, releasing divalent metals while consuming H^+^ ions (e.g., Oelkers et al., 2008). The consumption of H^+^ via silicate rock dissolution causes pH, HCO_3_^−^ and CO_3_^2‐^ concentrations to increase, resulting in the precipitation of carbonate minerals at a distance from the injection well. CarbFix1 verified these reactions by following the reaction progress of the dissolved injected CO_2_. Fluid samples were regularly collected and analyzed from the HN04 monitoring well. The use of non‐reactive tracers (SF_6_, SF_5_CF_3_) allowed CarbFix researchers to detect the arrival of the injection plume in HN04. The SF6 data from Phase 1 revealed an initial breakthrough peak in HN04 56 days after injection and the bulk arrival (peak concentration) 406 days after injection (Matter et al., 2016). Similarly, the conservative SF_5_CF3 tracer co‐injected with the Phase 2 CO_2_‐H_2_S gas mixture showed an initial breakthrough 58 days after initiation of the injection. The double peak of the tracer breakthrough curves is consistent with the pre‐injection tracer test results (Rezvani Khalilabad et al., 2008), suggesting a homogeneous porous media that is intersected by a low‐volume but fast flow fracture. Mass balance calculations for CarbFix1 using the carbon/conservative tracer ratios and the ^14^C/conservative tracer ratios indicated >95% loss of the dissolved CO_2_ via mineralization along the subsurface flow path from the injection to the monitoring well within 2 years (Figure 2; Matter et al., 2016). This was confirmed by corresponding mass balance calculations of dissolved Ca, Mg, Fe, and Si in the monitoring well fluids, mineral saturation states, as well as direct evidence of precipitated carbonate minerals (Snæbjörnsdóttir et al., 2017). Measured Ca, Mg, and Fe concentrations showed an initial increase during the injections with a gradual decline in the following months, which is consistent with the initial release of these elements from the basalt and their subsequent precipitation as carbonate minerals (Snæbjörnsdóttir et al., 2017).

A similar mass balance approach to determine the mass of CO_2_ and H_2_S fixed in the subsurface via mineralization was applied in CarbFix2. CarbFix2 researchers observed a faster mineralization rate compared to CarbFix1, where >50% of injected CO_2_ and 76% of sulfur were mineralized within just 4 to 9 months of Phase 1 (Clark et al., 2020). Furthermore, a twofold increase in the gas injection rate in CarbFix2 Phase 2, resulted in an increase in the mineralization to >60% for carbon and >85% for sulfur within 4 months (Clark et al., 2020). These calculations were based on the comparison of measured dissolved carbon and sulfur concentrations in the monitoring well fluids, with corresponding values determined by mass balance calculations based on conservative mixing (Gunnarsson et al., 2018). The enhanced rate of CO_2_ mineralization at CarbFix2 is suspected to be due to accelerated mineralization reactions at higher temperature, increased acidity of the injection fluids, and occurrence of fewer secondary minerals reaching supersaturation at the conditions of the injection zone (Clark et al., 2018).

#### 2.2.4 Next Steps

CarbFix1 and CarbFix2 successfully demonstrated subsurface mineralization of CO_2_ in basalt and created a new rapid methodology for the secure and permanent storage of CO_2_ through mineralization. Since the beginning of CarbFix1 in 2007, CarbFix as a subsidiary company of Reykjavik Energy further developed and upscaled this method, injecting >97,000 tons of CO_2_ into the subsurface at the CarbFix2 injection site. It is estimated that the porous basaltic rocks in Iceland can store ∼250 Gt of carbon as calcite (Gunnarsson et al., 2018). Additional upscaling of this methodology is, therefore, needed to accelerate mineralization as a global solution. For example, 1 million metric tons per year (a typical target of conventional sequestration) would require upscaling by a factor of about 100. Laboratory experiments using field samples to obtain critical reaction rates and thermodynamic data, and simulations aimed at optimizing CO_2_ mineralization processes in basalt, will help gain a more comprehensive understanding of the coupled subsurface processes. A possible limitation of the CarbFix project is its reliance on large quantities of freshwater, which makes significant upscaling challenging (only 5% of the injected mass at CarbFix was CO_2;_ Gislason & Oelkers, 2014). The next planned steps for CarbFix are to address this issue by testing the feasibility of dissolving CO_2_ into seawater, which is readily available at the site, before injection. Furthermore, researchers at CarbFix are working on combining direct air capture technologies with subsurface mineralization (Snæbjörnsdóttir et al., 2020).

### 2.3 The Wallula Basalt Pilot Demonstration Project

#### 2.3.1 Site Description

**Figure 3:**
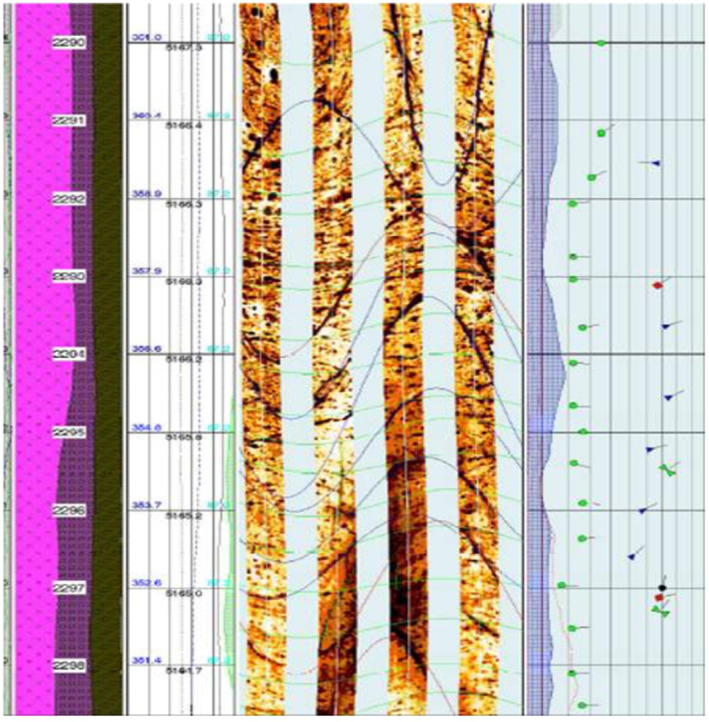
Fractures can be the primary flow paths for the passage and distribution of CO_2_ (dissolved or supercritical) within mafic and ultramafic rocks. This figure shows an image log depicting the natural tectonic fractures that appear as sinusoidal features within the Wallula Pilot Borehole. The green sinusoidal lines are interpreted as the flow features of the basalt. Characterization of the fracture network using tools such as image logs is an essential step in determining the storage potential of a reservoir. Image from McGrail, Schaef, et al. (2009).

The Wallula Basalt Sequestration pilot project was carried out in the southeastern region of Washington, USA, approximately 12 miles southeast of Pasco. Geologically, the site is hosted in the Miocene Columbia Plateau Province, a world‐class set of continental flood basalt deposits, with an estimated volume of more than 220,000 km^3^, covering over 320,000 km^2^ of Western Idaho, Oregon, and Washington (Reidel et al., 2002). In 2009, a borehole was drilled to a depth of 1,252 m, during which extensive surveys and hydrogeologic characterization of the flood basalts were carried out. A candidate injection zone was selected within the Grande Ronde Basalt lava flows, which is comprised of three permeable brecciated interflow zones (828−887 m) capped by an extremely low permeability member (∼10^−12^ to 10^−13^ m/s), which acts as a natural cap rock (McGrail et al., 2014). The mineralogy within these flows is, in order of abundance, plagioclase, augite, and volcanic glass, with secondary hematite, pyrite, zeolites, and clay minerals (Caprarelli & Reidel, 2004; McGrail, Sullivan, et al., 2009; Zakharova et al., 2012). Well cuttings indicated that the primary minerals filling vesicles, fractures, and veins are calcite and quartz (McGrail et al., 2011). The groundwater within the injection zone is classified as brackish, non‐potable, and sulfate‐rich, with elevated concentrations of fluoride (McGrail et al., 2011). Natural tectonic fractures are abundant in the borehole and are identified easily in image logs by their high dip sinusoidal features, which represent their primary depositional surfaces, as shown in Figure 3 (McGrail, Sullivan, et al., 2009). Other fractures are commonly in the form of cooling joints and short, irregular fractures that are perceived as impermeable (McGrail, Sullivan, et al., 2009). The pre‐injection reservoir conditions were ∼36°C and 7.7 MPa (McGrail, Schaef, Spane, Cliff, et al., 2017; McGrail, Schaef, Spane, Horner, et al., 2017).

#### 2.3.2 CO_2_ Injection and Post‐Injection Operations

In 2013, nearly one thousand metric tons (MT) of pure liquid CO_2_ was injected into the Grande Ronde Basalt flows over 25 days, averaging at a rate of ∼40 MT/day (McGrail, Schaef, Spane, Cliff, et al., 2017; McGrail, Schaef, Spane, Horner, et al., 2017). This carbon mineralization field demonstration was the first to inject CO_2_ as a supercritical fluid into continental flood basalts, eliminating the need to dissolve CO_2_ into large quantities of fresh water, as was done at CarbFix. To evaluate the extent to which the mineralization proceeded within the injection site, a suite of characterization techniques was used. Following the injection period until 2015, discrete groundwater samples were collected systematically at variable depths within the borehole for geochemical analyses (major cations, alkalinity, total dissolved solids (TDS)) to compare with pre‐injection concentrations (McGrail, Schaef, Spane, Cliff, et al., 2017; McGrail, Schaef, Spane, Horner, et al., 2017). Post‐injection wireline geophysical surveys and hydrologic tests were carried out to determine the extent, nature, and spatial distribution of CO_2_ within the reservoir and to assess changes in the hydrologic characteristics (McGrail, Schaef, Spane, Cliff, et al., 2017; McGrail, Schaef, Spane, Horner, et al., 2017). Before decommissioning the site in 2015, more than 50 side‐wall cores were collected from within the injection zone and subjected to detailed geochemical and geophysical analyses and 3D imaging to identify carbonates that precipitated from the injected CO_2_ (Polites et al., 2022).

#### 2.3.3 Key Observations

The collective results from the Wallula Basalt Pilot Project offer compelling evidence for the effectiveness of CO_2_ mineralization in continental flood basalts. Concentrations of major cations (e.g., Ca and Mg), alkalinity, and TDS measured in groundwater samples increased by 1.5–3 orders of magnitude from their pre‐injection formation concentrations, indicating the dissolution of CO_2_ into the groundwater and its reaction with the basalt's ferromagnesian minerals (McGrail, Schaef, Spane, Cliff, et al., 2017; McGrail, Schaef, Spane, Horner, et al., 2017). Geochemical calculations using the post‐injection groundwater data at reservoir conditions determined that the groundwater was supersaturated with respect to carbonate minerals such as calcite and dolomite (McGrail, Schaef, Spane, Cliff, et al., 2017; McGrail, Schaef, Spane, Horner, et al., 2017). Wireline logging characterization, including residual saturation and fluid temperature survey logging, identified highly electrically resistive free‐phase supercritical CO_2_ (scCO_2_) within the injection zone, which was not present prior to injection. It was estimated that 75%–90% of the pore water was replaced by CO_2_ in the middle layer, while ∼40% of the pore water was replaced in the bottom layer of the injection zone (McGrail, Schaef, Spane, Cliff, et al., 2017; McGrail, Schaef, Spane, Horner, et al., 2017). Simulations to determine the distribution of the CO_2_ within the formation yielded similar results (S. K. White et al., 2020).

While difficult to determine how CO_2_ was trapped within the injection zone at the time of sampling, the examination of sidewall cores identified two distinct carbonate phases that are interpreted to be formed from the injection, suggesting that some of the injected CO_2_ has already been mineralized (McGrail, Schaef, Spane, Cliff, et al., 2017; McGrail, Schaef, Spane, Horner, et al., 2017; Polites et al., 2022). The carbonate phases occurred as large nodules within the basalt vesicles and carbonate cements thinly coating the borehole wall face (McGrail, Schaef, Spane, Cliff, et al., 2017; McGrail, Schaef, Spane, Horner, et al., 2017). Analysis of the precipitates, including X‐ray diffraction, X‐ray microtomography (XMT), scanning electron microscopy, and energy dispersive X‐ray spectroscopy were carried out, and identified the carbonate nodules as the mineral ankerite, a Ca‐ and Fe‐rich carbonate mineral [Ca(Fe,Mg,Mn) (CO_3_)_2_]. These nodules were found to be chemically zoned, with a Ca‐rich phase near the center and a Fe‐rich phase at the surface (McGrail, Schaef, Spane, Cliff, et al., 2017; McGrail, Schaef, Spane, Horner, et al., 2017), with a minor zonation in Mn decreasing toward the surface (Polites et al., 2022). The zonation within the nodules is suspected to be due to temporary changes in the formation water's geochemistry (oxygen fugacity) due to CO_2_ injection, prior to its buffering by the surrounding basaltic rock. These findings are distinct from analyses on naturally occurring calcite veins within the sidewall, which had no trends in major cations (McGrail, Schaef, Spane, Cliff, et al., 2017; McGrail, Schaef, Spane, Horner, et al., 2017). Selected samples were thin‐sectioned for a more thorough investigation of the mineralogy and spatial and paragenetic relationship of the minerals (Polites et al., 2022). A fibrous texture was observed within the nodules, suggesting that the ankerite phase was a result of the pseudomorphic transformation of aragonite due to increased Mn^2+^ and Fe^2+^ (Polites et al., 2022).

Comparative isotopic characterization of the post‐injection ankerite nodules, post‐injection groundwater samples, preexisting natural calcite veins, and the injected CO_2_ offers the most convincing evidence of CO_2_ mineralization within the Columbia River basalt formation as a result of the pilot test. Using nano secondary ion mass spectrometry (nanoSIMS) to measure delta oxygen‐18 (δ^18^O) and delta carbon‐13 (δ^13^C) concentrations (McGrail, Schaef, Spane, Cliff, et al., 2017; McGrail, Schaef, Spane, Horner, et al., 2017), the measured values for the ankerite nodules (δ^13^C = −37.7 ± 2.19‰, δ^18^O = −22.5 ± 2.38‰), post‐injection formation water (δ^13^C = −32.2 ± 0.79‰, δ^18^O = −22.3 ± 1.53‰), and CO_2_ (δ^13^C = −36.3 ± 0.09‰, δ^18^O = −27.9 ± 0.51) indicate a common source, distinct from the natural calcite values (δ^13^C = 15.8 ± 1.01‰, δ^18^O = −20.0 ± 0.41) (McGrail, Schaef, Spane, Cliff, et al., 2017; McGrail, Schaef, Spane, Horner, et al., 2017). Based on the data derived from the multitude of tests, numerical modeling estimates that ∼60% of the supercritical CO_2_ was mineralized during the first two years following injection, with the carbonate precipitation encompassing ∼4% of the reservoir's available pore space (S. K. White et al., 2020). Based on these calculations, the total mineral trapping capacity of the Grande Ronde Basalt is estimated at 47 kg of CO_2_/m^3^ (Xiong et al., 2018). Assuming the carbonation rate were to remain constant, and the pore pressure was fixed at ∼4%, it would, thus, take 38 years to fill the pore space in the Grande Ronde Basalt at 100°C (Xiong et al., 2018).

#### 2.3.4 Role of Fractures

Conclusions specific to the spatial distribution of the CO_2_ mineralization within the fracture network of the Wallula basalt injection zone are absent from the literature. Aside from the initial fracture characterization within the borehole, the orientation of the fracture network within the basalt is not defined. Therefore, it is difficult to assess the role of fractures in the distribution and reaction of CO_2_ within the reservoir. Furthermore, Columbia River basalt flows are highly heterogeneous and generally consist of dense flow interiors sandwiched by irregular, brecciated, and vesicular flow tops and bottoms (Burns et al., 2015). Therefore, fractures will not be the only source of fluid flow and storage volume. Nevertheless, models can shed light on the mineralization potential of a system, as exemplified in a recent simulation study, which used ensemble simulation methods to predict the geomechanical integrity of the Wallula Basalt Project reservoir (Jayne et al., 2019). This study provides a first order estimate for the potential evolution of the permeability of the reservoir during CO_2_ injection at a large scale. The authors simulated the injection of CO_2_ into the reservoir at a constant mass of 21.6 kg/s and variable permeability distributions. Notably, they determined that the pressure build‐up near the injection site due to CO_2_ injection geomechanically impacts a significantly larger radius than the injection fluid itself (Jayne et al., 2019). This pressure build‐up could result in shear slip of pre‐existing fractures, which can ultimately increase the total permeability of the reservoir (Jayne et al., 2019). It should be noted that this model does not consider geochemical reactions, where dissolution and precipitation could alter the aperture of the fractures. Given the heterogeneous nature of basalt reservoirs, extensive characterization is necessary to provide an accurate understanding of the storage potential and the evolution of the fracture network as CO_2_ is injected and mineralized. Moreover, it is important to understand how the injection of scCO_2_ versus CO_2_ dissolved in water will influence the reactivity and storage potential of a fractured reservoir. Ultimately, future research should emphasize the geomechanical evolution of the reservoir, in addition to changes in the geochemistry.

### 2.4 Subsurface Mineralization in Peridotite—Oman Drilling Project

Peridotite is a major component of the Earth's upper mantle and is exposed at the earth's surface as ophiolite massifs, of which the largest is the Oman ophiolite in Oman and the United Arab Emirates (e.g., Kelemen & Matter, 2008). Peridotite is mainly composed of the primary minerals olivine, pyroxene, and spinel, which are commonly partially or completely altered to mixtures of serpentine, brucite, Fe‐oxides, and oxyhydroxides by water‐rock reactions (e.g., Kelemen et al., 2019). Extensive natural CO_2_ mineralization has been observed in peridotite, with predicted rates on the order of 1,000 t CO_2_/km^3^/yr (Kelemen et al., 2011; Mervine et al., 2014). At Oman, 5,000 tons of carbonate is estimated to form within the Oman peridotite each year (Kelemen et al., 2011; Kelemen & Matter, 2008). Listvenites―fully carbonated peridotites― are exposed at the surface of the Oman ophiolite, having formed ∼90 million years ago at temperatures of ∼80–130°C, depths of 10–50 km, and under elevated pressure (*P*_CO2_ ∼1–5 bars) (Beinlich et al., 2020; Falk & Kelemen, 2015; Kelemen, Matter, et al., 2020; Kelemen, McQueen, et al., 2020). These listvenites offer a natural analog to present‐day carbon mineralization and insight into the capacity of these rocks to store CO_2_.

The Oman Drilling Project (OmanDP), an International Continental Scientific Drilling Program (ICDP) project, was established to improve our quantitative understanding of processes of mass and energy transfer between the mantle, crust, hydrosphere, atmosphere, and biosphere. The project drilled a cross‐section through the Oman ophiolite from 2016 to 2019, where a total of 5,400 m (15 boreholes) were drilled and 3,220 m of core collected. Included in this project was the establishment of the Multi‐Borehole Observatory (MBO) to investigate the geochemical, hydrological, and geomechanical alteration of partially serpentinized peridotite and study active low‐temperature alteration processes, including carbon mineralization. In contrast to the CarbFix and Wallula projects, CO_2_ mineralization studies in Oman are still in their early stages. Nonetheless, a suite of fracture and geochemical parameters have been collected as an initial stage for reservoir characterization, as described below.

#### 2.4.1 MBO

The OmanDP MBO in the Wadi Tayin massi of the Oman ophiolite, SE of the capital Muscat, includes four air‐rotary‐drilled boreholes and three diamond‐cored boreholes. The total depth of the boreholes ranges from 300 to 400 m (Kelemen, Matter, et al., 2020; Kelemen, McQueen, et al., 2020). The boreholes penetrate two major rock sequences: fully serpentinized dunites with pyroxenite dikes and 80%–100% serpentinized harzburgite with gabbro (Kelemen, Matter, et al., 2020; Kelemen, McQueen, et al., 2020). The boreholes and rock sequences were fully characterized using wireline geophysical logging and hydrogeological testing to investigate the natural carbonation process.

#### 2.4.2 Key Findings

**Figure 4:**
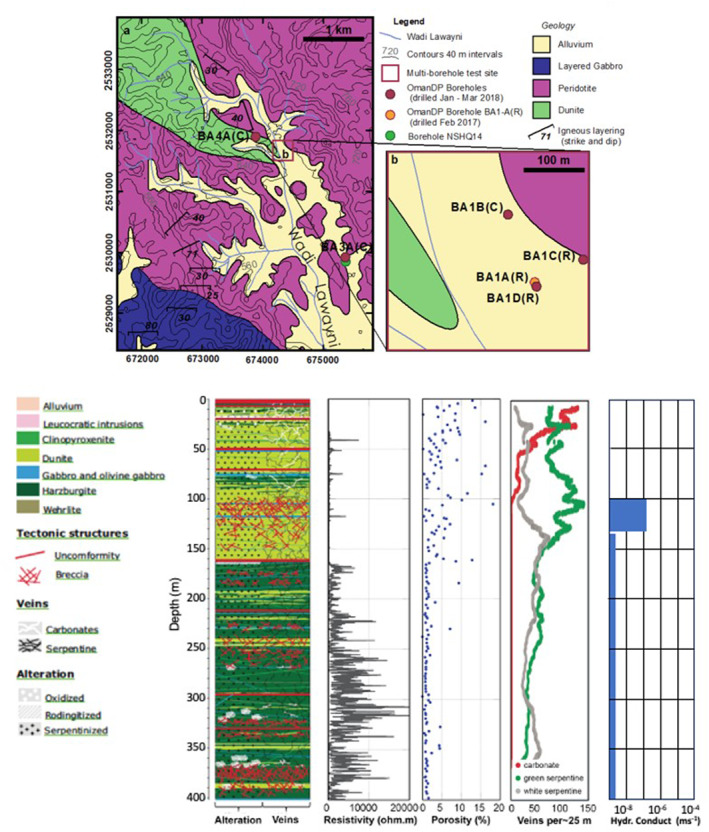
Geophysical borehole and core logs from the Oman Drilling Project reveal a pervasive fracture and vein network. Data collected from borehole BA1B (as shown on the map) includes a lithostratigraphy log (OmanDP Multi‐Borehole Observatory), wireline borehole resistivity log, and downhole plots of discrete sample measurements of porosity and vein types (25‐m average; Kelemen, Matter, et al., 2020; Kelemen, McQueen, et al., 2020) as well as a summary of the estimated hydraulic conductivity of discrete intervals based on pumping tests (Lods et al., 2020). The data shows that the permeability decreases with depth, relating to a decrease in alteration and crack/vein density (Kelemen et al., 2021).

Geophysical borehole and core logs reveal a pervasive fracture and vein network, primarily filled with secondary minerals, and a rock matrix with micro to nanopores (Kelemen, Matter, et al., 2020; Kelemen, McQueen, et al., 2020). The serpentinized dunite sequence has higher porosity, higher fracture density, and lower electrical resistivity than the serpentinized harzburgite sequence (Figure 4). Permeability decreases with depth and ranges from 10^−12^ m^2^ at <50 m to <10^−14^ m^2^ at <150 m, and <10^−17^ m^2^ below 150 m depth (Lods et al., 2020). Decreasing permeability with increasing depth correlates with decreasing alteration and decreasing crack/vein density (Kelemen et al., 2021). Decreasing permeability also correlates with the type of groundwater that is present in the peridotite. A Mg‐HCO_3_, pH ∼ 8 oxidized groundwater occurs in the highly weathered and higher permeability zone near the surface, whereas a Ca‐OH‐rich, pH > 11 and highly reduced groundwater is found at a greater depth, thought to be associated with more extensive and deeper interaction between peridotite and groundwater and a reduction of Mg and CO_2_ due to the mineralization of carbonate minerals and serpentine (Kelemen et al., 2021). Borehole flowmeter logs and multi‐level cross‐hole hydraulic experiments show that complex vertical and horizontal structural heterogeneities govern fluid flow (Lods et al., 2020). However, fluid pathways in the fracture network and their interconnectivity are poorly characterized and understood.

The present‐day carbonation of peridotite is driven by near‐surface weathering, as indicated by the rapidly decreasing occurrence of Ca‐ and Mg‐carbonate‐filled veins with depth. No carbonate veins were found in the MBO cores below 100 m (Kelemen et al., 2021). This agrees with the proposed reaction path of infiltrating surface water and descending shallow groundwater through the upper, high‐permeability aquifer with progressive interaction with peridotite along the flow paths (Dewandel et al., 2005; Paukert et al., 2012). This implies that the deeper subsurface has a greater CO_2_ mineralization potential that could be utilized for engineered carbon mineralization. Indeed, the injection of CO_2_‐rich fluids into peridotite at depth below the weathering horizon could enable CO_2_ mineralization, potentially storing 10^5^ to 10^8^ GtCO_2_ (Kelemen & Matter, 2008). However, new data from the MBO site reveals that injectivity into the deeper subsurface is greatly limited by the low permeability of the peridotite. Thus, engineered carbon mineralization in peridotite will most likely require permeability enhancement via industrial hydraulic fracturing or naturally induced subcritical fracturing and reaction‐driven cracking/damage due to mineralization. The latter depends on the increase in differential stress in the rock by the solid‐volume increasing carbonation reaction, resulting in the generation of fractures and subsequent increase in the rock's permeability and reactive surface area (See Section 3; e.g., Jamtveit et al., 2008; Kelemen & Hirth, 2012; Uno et al., 2022; W. Zhu et al., 2016).

#### 2.4.3 Next Steps

Experiments at different length scales from mineral grain to field scale are required to better understand the coupled thermo‐hydro‐mechanical‐chemical (THMC) processes in fracture‐dominated systems, such as peridotite (as discussed in the next section) (Plümper & Matter, 2023). Like the CarbFix project, the technology start‐up 44.01, in collaboration with industrial and academic partners, started multiple research‐to‐application initiatives to demonstrate the feasibility of carbon mineralization in peridotite (www.4401.earth). Project Chalk was established to test CO_2_ mineralization in peridotite at the MBO site in Oman. Utilizing MBO boreholes, several hundred kilograms of CO_2_ dissolved in water have been injected and the progress of mineralization is being monitored. In addition, a mineralization pilot test site in Fujairah (UAE) successfully injected 10 tonnes of air‐captured CO_2_ into peridotite in 2023, with plans to scale up this operation to 300 tonnes soon (www.4401.earth).

### 2.5 Challenges With Industrial‐Scale Carbon Mineralization

Field experiments, such as the CarbFix, Wallula, and Oman projects, have successfully demonstrated the feasibility of in‐situ mineral carbonation in mafic and ultramafic rocks. For mineralization to be a global solution, significant upscaling and acceleration of the deployment of mineralization field sites are needed. This will require maximizing CO_2_ injection rates per borehole, sustaining long‐term injection and fluid flow rates in the storage reservoir, and maximizing dissolution and carbonate precipitation rates. Challenges remain from the pilot projects, as in many cases detailed hydrological, chemical, and mechanical parameters cannot be derived from field observations. Outstanding questions include: (a) how to minimize the requirement of large volumes of fresh water for dissolved CO_2_ injection; (b) the feasibility of supercritical CO_2_ injection, which will require a caprock and perhaps longer retention times in the reservoir to fully mineralize the injected CO_2_; (c) constraining the timescales of the different processes, including mineral dissolution and precipitation rates; and (d) defining reactive surface area, reactions and rates of reaction in fractures (advection‐dominated) and in pores & matrix (diffusion‐dominated). In addition to field characterization data, laboratory experiments, discussed in the next section, will be critical for addressing these outstanding questions, and deriving important parameters required for modeling CO_2_ mineralization.

## 3 Experimental Studies on Carbon Mineralization in Fractured Systems

### 3.1 Introduction

Observations derived from pilot‐scale carbon mineralization field tests delineate important knowledge gaps that can be better understood through controlled laboratory experiments. These knowledge gaps center on the overarching question of the scalability and feasibility of large‐scale CO_2_ mineralization in mafic and ultramafic rocks, which requires an understanding of reaction rates, feedbacks between geochemical and geomechanical reactions, and the influence of flow and transport, among other factors. Recent advances in experimental capabilities, such as high pressure (P)/temperature (T) systems, coupled stress, flow and reaction in geomaterials, and in situ imaging capabilities, have aided in the derivation of parameters needed to model mineralization processes and field site development. In this review, we highlight four key areas of laboratory research that are centered on bridging these knowledge gaps: (a) reactivity of mafic minerals in CO_2_‐rich environments (Section 3.2), (b) the dynamic interaction between fluid flow and chemical reactions in fractures (Section 3.3), (c) the coupling between fluid flow and mechanics for stimulation (Section 3.4), and (d) the coupled feedback between chemical reactions and the development of fractures (Section 3.5).

*Reactivity of the minerals*: CO_2_ mineralization in mafic and ultramafic rocks is induced by the injection of CO_2_ or CO_2_‐charged water, dissolution of key divalent cation‐bearing minerals (olivine, pyroxene, serpentine, plagioclase, volcanic glass), and the precipitation of a variety of carbonates including magnesite (MgCO_3_), calcite (CaCO_3_), siderite (FeCO_3_), and ankerite (Ca(Mg,Fe) (CO_3_)_2_) (Romanov et al., 2015). These reactions occur in an acidic aqueous medium produced by high‐pressure CO_2_, where the pH is controlled to first order by equilibrium between CO_2_ and the precipitating carbonates. The extent of carbonation is controlled by the dissolution rates, precipitation rates, and the available surface area (Romanov et al., 2015), which are governed by many factors including hydrodynamic effects, fracture characteristics, surface passivation, and reservoir geochemistry.

*Fluid flow and chemical reactions in fractures:* A major challenge in optimizing in situ carbon mineralization arises from the fact that the process depends not only on the mineral distribution and geochemistry, but also on the structural properties of the fractures, and the rock matrix (aperture, reactive surface area, porosity), fluid flow conditions (flow rate), and fluid properties (density, viscosity) (Deng, Molins, et al., 2018; Deng, Steefel, et al., 2018; S. Li et al., 2007). Furthermore, dissolution and precipitation reactions can simultaneously create or destroy permeable paths of fluid flow, which are often dominated by complex fracture networks in mafic and ultramafic rocks (Jiménez‐Martínez et al., 2020; Luquot & Gouze, 2009; Noiriel & Soulaine, 2021; Noiriel et al., 2013; H. Yoon et al., 2019). This fluid flow is required for the dissolution of a rock matrix and delivery of chemical species (CO_2_, cations) needed to mineralize carbon; however, those mineralization products can also clog flow paths. These interconnected feedbacks demonstrate how CO_2_‐fluid‐rock reactions under fluid flow are highly nonlinear and dynamic.

*Fluid flow and mechanics for stimulation*: Permeability enhancement, such as hydraulic fracturing and hydro‐shearing, may be needed to artificially stimulate fracture growth in mafic and ultramafic rocks for sustained, extensive CO_2_ mineralization. Two primary methods of permeability enhancement in deep subsurface rocks include hydraulic fracturing, which requires fast‐rate fluid injection to create new fractures, and hydro‐shearing, which involves moderate‐rate fluid injection to reactivate pre‐existing geological discontinuities. Both hydraulic fracturing and hydro‐shearing have been extensively studied in hydrocarbon‐bearing sedimentary rocks (Bunger et al., 2005; Detournay, 2016; Fisher & Warpinski, 2012; W. Li et al., 2021) and geothermal rocks (Meng et al., 2022; Rinaldi & Rutqvist, 2019; Yuan et al., 2020), but rarely in CO_2_‐reactive mafic and ultramafic rocks (Nicolas & Jackson, 1982).

*Coupled feedback of chemical reactions and fracture development*: Efficient carbonation of basalt and peridotite requires widespread porosity and/or an extensive fracture network through which the injected CO_2_ can flow and react with the host rock. Basalt formations naturally contain more porosity and fractures than peridotite due to porous inter‐flow regions and thermally fractured flow boundaries (McGrail et al., 2006). However, carbonating a large mass of impermeable basalt in flow centers or impermeable peridotite with a porosity of about 1% (Kelemen et al., 2011) requires some form of anthropogenically‐ or tectonically‐induced fracturing. It is possible that mineral carbonation itself may provide the means to access more reactive surfaces, either through reaction‐driven fracturing or subcritical fracturing. These dynamics must be better understood to determine the long‐term feasibility of carbon storage in mafic and ultramafic rock.

Whether experimental observations hold in the field (Section 3.6) is of critical importance for furthering field site testing and providing realistic parameters for modeling studies. Although experiments cannot inform large‐scale processes, they play an important role in constraining kinetic rates, thermodynamic data, and small‐scale fracture properties. While carbonation beyond a fracture surface has been shown to occur in nature (Kelemen et al., 2011), as discussed in previous sections, it is critical to demonstrate that this process can be engineered for large‐scale, long‐term anthropogenic CO_2_ mineralization operations. Here we review the experimental evidence that can provide insight into the key processes that occurred (and cannot be directly observed) during the CO_2_ mineralization operations discussed above. Findings from these studies, such as mechanisms and timescales, are critical to constrain models (as discussed in the next section) and provide insights that will aid in optimizing anthropogenic CO_2_ injection into mafic and ultramafic rocks.

### 3.2 Reactivity of Mafic Minerals

Field‐scale carbonation efficiency is in large part defined by the individual reactivities of constituent rocks and minerals toward carbon mineralization, which are typically determined using batch experimental studies. The work of Gadikota et al. (2020) measured the reactivity of common mafic minerals and rocks in NaCl− and NaHCO_3_−bearing solutions including Mg‐rich olivine, labradorite plagioclase, and basalt, and found that the extents of carbonation were 85%, 35%, and 9%, respectively, under optimized conditions. This finding has important implications for assessing the relative advantages of peridotite and basalt: peridotites are rich in olivine, which has the most favorable carbonation kinetics; and basalts are rich in labradorite plagioclase with less favorable kinetics and the additional potential to leave greater residual barriers (clay) to continued carbonation.

The overall extent of mineralization will depend on (a) the dissolution of mafic minerals, which promotes the release of divalent cations and (b) the rate of carbonate mineral precipitation. Dissolution is thus an essential first step in the mineralization process. Of the possible reactant minerals, the dissolution rate of olivine has received extensive consideration due to its higher reactivity (Oelkers et al., 2018; Rashid et al., 2022). The review of Oelkers et al. (2018) concluded that the primary factors that control olivine dissolution rates are temperature, pH, water activity, and surface area. In addition, the degree of serpentinization of olivine is also expected to affect the rates (Klein & Garrido, 2011; Klein & McCollom, 2013). The dissolution rates of other key minerals such as plagioclase (De Obeso et al., 2023; Gudbrandsson et al., 2014; Min et al., 2015), pyroxene (Golubev et al., 2005; Knauss et al., 1993), and serpentine (Thom et al., 2013) are also reported in the literature and summarized in Kelemen et al. (2019). However, the dissolution rates for olivine and other mafic minerals vary significantly among experimental studies (Oelkers et al., 2018), and thus, further research is required to more tightly constrain realistic dissolution rates.

In contrast to dissolution rates, few experimental efforts to constrain precipitation rates have been conducted. There is a framework for calculating rates of precipitation by extrapolating dissolution data (transition‐state‐theory; e.g., Lasaga, 1981). However, many experiments show precipitation rates that are a complex function of supersaturation and pH conditions, with particular sensitivity to other solution components (Shiraki & Brantley, 1995; Teng et al., 2000; Y. Zhang & Dawe, 2000). Furthermore, while calcite precipitation is relatively well understood (Dreybrodt et al., 1997; Inskeep & Bloom, 1985; Y. Zhang & Dawe, 2000), magnesite precipitation is more complicated, with different controls and greater kinetic barriers (Giammar et al., 2005; Hellevang et al., 2013), usually attributed to freeing magnesium ions from a strong hydration shell (Power et al., 2017). Giammar et al. (2005) determined that supersaturation indices of 0.25–1.14 were required to induce magnesite precipitation and growth. Moreover, experimental studies have shown that the rate of magnesite precipitation from olivine dissolution is quite slow at temperatures ≤90°C (Gadikota et al., 2014), and that magnesite is unlikely to form at temperatures ≤75°C (Oelkers et al., 2018). The surface area on which precipitation may occur is even more poorly constrained, as precipitation on solid surfaces (heterogeneously), in solution (homogeneously), and/or only on particular faces of certain crystals are all possible options. Therefore, case‐specific measurements of precipitation rates are needed at various conditions relevant to reservoirs proposed for CO_2_ storage. At this time, there is not a well‐accepted kinetic model that predicts the rate of magnesite or other carbonate precipitation from olivine or other mafic minerals.

As previously mentioned, when comparing the carbonation potential of peridotites and basalts, it is important to consider the overall geochemical makeup. Peridotites are composed of more than 90% Mg compared to Ca and Fe (Kelemen et al., 2011), while basalts contain subequal amounts of Mg, Ca, and Fe, generally with Fe > Ca > Mg (Schaef et al., 2009). Experiments by Schaef et al. (2009) demonstrated that carbonate precipitation in basalt had complex zonation that generally varied from dominantly Ca‐rich to Fe‐rich, with less than 20% Mg. This suggests that the carbonation of basalt does not suffer from the same kinetic limitations of magnesite precipitation, as seen in peridotite, thanks to the presence of Ca and Fe as alternative divalent cation reactants. While these findings may appear contradictory to those of Gadikota et al. (2020), which showed that the extent of carbonation of olivine was significantly greater than that of basalt, the basalt reactivity experiments in Schaef et al. (2009) were conducted at lower temperatures and CO_2_ pressures (60–100n °C, 10.34 MPa CO_2_). Additional comprehensive studies comparing the mineralization potential of basalt versus peridotite at lower temperatures are needed to determine whether high‐temperature trends hold. Other factors contributing to the reactivity differences between these two rocks include the potential for higher divalent cation concentrations in peridotite due to its high olivine content and the generally higher porosity and permeability of basalt, which may aid in more widespread mineralization under fluid flow conditions.

Despite variable results of laboratory‐based dissolution measurements, the principal uncertainty in the field is likely to be the available surface area where the chemical reactions take place. For a fracture system in an impermeable rock, a conservative assumption would be the geometric fracture surface area. However, Van Noort et al. (2013) provided experimental evidence to suggest that the effective reactive surface area could be 100 times greater than the geometric fracture surface due to grain‐scale structure, even in nominally impermeable peridotite. Whether this apparent surface area would persist as the reaction front penetrates deeper into the peridotite is unknown. Nonetheless, compared with the mass of the bulk rock and considering reaction rates determined on fine‐grained powders, this is a relatively small surface area that will limit the net production of divalent cations needed for carbonation.

Even with the uncertainties in dissolution and precipitation rates, basalts and peridotites possess enormous theoretical carbonation capacities (Matter & Kelemen, 2009; McGrail et al., 2006). As discussed, batch experimental studies of dissolution and precipitation show that reaction rates are relatively slow, meaning that uncertainty in kinetic data plays a significant role in constraining the timescale of mineralizing CO_2_ in basalt and peridotite sequestration projects. Further experimental studies must be designed to better constrain rates and extents of carbonation of whole rock samples. In the following two sections, we examine our current understanding of the potential role of coupled processes in accelerating mineralization, including the coupling of fluid flow and reaction and the coupling of flow, reaction, and mechanical processes as important considerations in mineralization efficiency.

### 3.3 Coupling Between Fluid Flow and CO_2_ Mineralization Reactions

For successful prediction and optimization of mineralization, it is necessary to identify the key regimes of coupled dissolution and precipitation behavior as a function of the main parameters of geochemistry, flow, and rock structure. Carefully designed laboratory experiments play an essential role in understanding mineralization because they allow for the isolation of key processes of interest and enable the detailed characterization of rocks undergoing carbonation.

Experimental studies have elucidated important interface‐coupled dissolution and precipitation processes that are relevant to carbon mineralization (Cubillas et al., 2005; Raza et al., 2022; Ruiz‐Agudo et al., 2014). Multiple coupled dissolution and precipitation regimes exist: at one extreme is surface passivation, where mineral precipitation results in coatings that limit reactive surface area and inhibit reactions, while at the other extreme, complete mineralization occurs, resulting from net increases in porosity and sustained reaction (Forjanes et al., 2020). A main challenge, however, is that most studies are conducted under well‐mixed batch conditions (Løge et al., 2023; Xiong & Giammar, 2014; Xiong, Wells, Menefee, et al., 2017), with limited studies that honor realistic fluid flow conditions (Baek et al., 2019; Menefee et al., 2017, 2018). It is well established that hydrodynamic conditions strongly impact reaction kinetics and dissolution and precipitation patterns (Arvidson et al., 2003; Colombani, 2008; Osselin et al., 2016; Qin & Beckingham, 2021; Yang et al., 2024), but there remain open questions on how best to utilize the parameters derived from batch experiments in carbon mineralization. In geologic fractured porous media systems, variations in reaction rates and reactive surface areas lead to highly heterogeneous dissolution and precipitation patterns and mineralization rates that are significantly different from batch conditions (L. Li et al., 2008; M. Liu et al., 2022). For example, studies of dissolution under flow have demonstrated how the relative rates of reaction and transport, codified by the Dakmköhler number, dramatically alter spatiotemporal trends of dissolution and effective dissolution rates (Fredd & Fogler, 1998; Golfier et al., 2002). These trends should also manifest in coupled dissolution‐precipitation systems. As a result, we cannot currently predict CO_2_ mineralization rates under relevant flow conditions.

Recent laboratory core flow studies conducted under reservoir pressures and temperatures have provided valuable insights into the interplay of flow, transport, and geochemistry on CO_2_ mineralization (Adeoye et al., 2017; Andreani et al., 2009; Luhmann, Tutolo, Bagley, et al., 2017; Luhmann, Tutolo, Tan, et al., 2017). For example, Luhmann, Tutolo, Bagley, et al. (2017) and Luhmann, Tutolo, Tan, et al. (2017) found that lower flow led to greater secondary mineralization and consequently lowered the rock permeability. Andreani et al. (2009) found similar results and concluded that moderate injection rates are optimal, ensuring partial carbonation while maintaining the permeability of the reservoir. The pH level of the fluid has also emerged as a pivotal factor influencing the dissolution‐precipitation dynamics and carbonation efficiency (Luhmann, Tutolo, Tan, et al., 2017; Menefee et al., 2018; Xiong, Wells, & Giammar, 2017; Xiong, Wells, Menefee, et al., 2017). Lower pH values favor dissolution over precipitation, resulting in a net increase in porosity. In contrast, higher pH fluids suppress dissolution and promote rapid carbonate precipitation. The role of fluid flow further influences these dynamics (Menefee et al., 2018; Phukan et al., 2021). Elevated flow rates, typically observed in fractures, result in enhanced dissolution due to the high flux of low pH fluid that creates slow precipitation kinetics compared to transport (i.e., low Dahmköhler number). Conversely, stagnant zones, where diffusion controls transport, have a low flux of acidic fluid, resulting in higher pH values and rapid precipitation. While these overall trends in precipitation and dissolution regimes in carbon mineralization are understood, many questions remain in constraining the dominant regimes as a function of flow and geochemical parameters.

**Figure 5:**
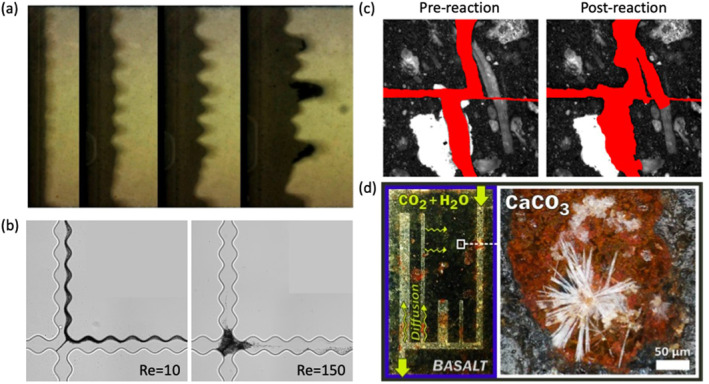
Laboratory experiments can help visualize fluid flow and reaction and identify key processes that govern carbon mineralization. Some recent examples include: (a) Investigating reactive‐infiltration instability to understand advection, diffusion, and reaction in an analog fracture. The direction of water infiltration into gypsum chips is from the left to right. The dissolved portion of the chip is indicated by a dark color, while the undissolved portion is shown in a light color (Osselin et al., 2016). (b) Visualization of fluid inertia effects on mineral precipitation patterns in microfluidics experiments. Barium and sulfate solutions are co‐injected into the intersection, where they are subsequently mixed and precipitates are formed. The barite precipitates are indicated in black (Yang et al., 2024). (c) Characterizing mineral mechanistic processes during the dissolution of natural rock samples embedded in microfluidic cells. The fracture space is indicated in red, while the surrounding rocks are depicted in black and white (Ling et al., 2022). (d) Understanding how temperature, chemistry, and transport limitations affect mineral dissolution and precipitation in flow‐through rock core experiments. Carbonation occurs within the diffusion‐limited zone when CO_2_‐charged water is injected into a fractured basalt rock sample (Menefee et al., 2018).

Laboratory experiments that visualize fluid flow and mineral dissolution and precipitation shed light on key interacting processes governing carbon mineralization (Figure 5). In particular, experiments using lab‐on‐a‐chip (LoC), a microfluidic platform that allows detailed spatiotemporal characterization of micro‐scale phenomena, show great promise (Datta et al., 2023). These experiments effectively reproduce complex features of fractured porous media structures that are central to efficient mineralization, such as fractures and the surrounding rock matrix, which are needed to sustain both flow and mineralization (Cardona & Santamarina, 2023; Ghassemi & Suresh Kumar, 2007; Kong et al., 2023; Porter et al., 2015; Steefel & Lichtner, 1994; Q. Zhang et al., 2022). The LoC approach can also realize and characterize a wide range of fluid flow conditions. For example, this platform can visualize often‐neglected inertial laminar flows that readily occur in fractured media. Inertial laminar flows fall between the well‐known extremes of creeping (Stokes) and turbulent flow regimes. They are distinct in that they have more complex steady flow structures, such as helical flows and recirculating flows (weak inertia), or periodic flow structures, like vortex shedding (strong inertia), which are not observed in creeping flows. However, they do not exhibit the chaotic behavior that is characteristic of turbulent flows (Wood, 2007; Wood et al., 2020). Recent studies, including LoC experiments, have shown that inertial laminar flow regimes, even comparatively weak ones, strongly affect reactive transport and the patterns of mineralization and dissolution, and may contribute to the variation in observed reaction rates (Figure 5b) (Crevacore et al., 2016; Deng, Molins, et al., 2018; Deng, Steefel, et al., 2018; Lee & Kang, 2020; M. Liu et al., 2020; Ma et al., 2021; Moosavi et al., 2019; Yang et al., 2024; S. Yoon & Kang, 2023). In another example, density‐driven flows are also expected to occur during carbon mineralization due to density contrasts arising from the injection of higher‐density CO_2_‐charged water into groundwaters (Snæbjörnsdóttir et al., 2020; Z. Xu et al., 2023) or mineral dissolution, which will cause unexpected instabilities and distinctive fracture permeability scaling laws (Ahoulou et al., 2020; H. Cao et al., 2023; Huang et al., 2020; J. Zhu & Cheng, 2018). Laboratory experiments, especially those using the LoC approach to visualize these complex fluid flow and transport processes in fractured porous media, have great potential to interrogate these interlinked flow and geochemical processes.

More advanced experiments coupling fluid dynamics and geochemical processes are required to address questions relevant to carbon mineralization. In particular, the geochemical properties of geologic media must be captured to investigate coupled dissolution and precipitation processes (Figures 5c and 5d). There are two major directions in this effort: experiments in rock‐analog systems (Jones & Detwiler, 2019; Neuville et al., 2017; Osselin et al., 2016; Park et al., 2021; Poonoosamy et al., 2020; H. Yoon et al., 2019) and embedding real rock samples into microfluidics or other flow‐through setups (Deng et al., 2020; Fazeli et al., 2020; Ling et al., 2022; Neil et al., 2024; Singh et al., 2017). Using rock‐analog systems (Figure 5a), one can efficiently explore wide ranges of fluid flow and reaction regimes with direct visualization at more feasible time scales. Such experiments enable the identification of the various regimes of dissolution‐precipitation reactions that occur during mineralization, including a coupled regime, where co‐located dissolution and precipitation result in sustained mineralization, a passivation regime, where precipitation rates are so fast that the reaction shuts down, and a decoupled regime, where precipitation occurs in a location far from the initial dissolution. Recent advancements have also enabled high‐pressure‐high‐temperature microfluidics and core flooding systems, which are important for realizing in situ reservoir conditions during carbon mineralization of mafic/ultramafic rocks with direct visualization (Jiménez‐Martínez et al., 2020; Menefee et al., 2018; Porter et al., 2015). Despite these advances, the slow dissolution kinetics of mafic and ultramafic rocks have resulted in either dissolution‐dominated or precipitation‐dominated processes depending on the injection fluid chemistry (Luhmann, Tutolo, Tan, et al., 2017). For the purposes of capturing the spatial and temporal evolution of both dissolution and precipitation processes, microfluidic and core flooding experiments must be carried out under high pressure and temperature conditions with carefully tuned experimental parameters, including fluid chemistry and residence time of fluids injected.

By combining these systematic laboratory experiments with validated models and reactive transport simulations (Section 4.5), key dimensionless numbers that can help delineate major carbon mineralization regimes may be identified. These include Damköhler numbers that compare reaction rates with transport rates, Peclet numbers that compare advective and diffusive mass transfer, and Reynolds and Rayleigh numbers that quantify inertia and density‐driven flow, respectively. Dissolution‐precipitation reactions are largely influenced by the dominant minerals, such as olivine in peridotite and plagioclase feldspars in basalt (Kelemen et al., 2019). The kinetics of these reactions have been extensively characterized in batch experiments (Pokrovsky & Schott, 2000; Schaef & McGrail, 2009). Consequently, the controlling Damköhler values can be determined based on the representative minerals for dissolution and precipitation. Regarding nucleation, it typically governs the early stages of mineral precipitation. Therefore, the overall precipitation kinetics are primarily controlled by crystal growth (Lasaga, 2014). However, depending on the distribution of minerals, spatially heterogeneous nucleation sites could significantly influence precipitation patterns and clogging. Therefore, studying the effects of nucleation on mineral precipitation is an important area of research.

The improved understanding of mineralization processes under fluid flow conditions could lead to improved engineering strategies to overcome reaction slowdowns, such as creating new fracture surfaces and flow paths by controlling injection strategies. Additional well‐controlled core flooding experiments of real rock at high‐pressure‐high‐temperature conditions and nanoscale characterization of dissolution and precipitation phenomena (e.g., etch‐pits, nucleation) are needed to verify results obtained from LoC experiments (Kim et al., 2021; Luhmann, Tutolo, Tan, et al., 2017; Noiriel et al., 2020). Ultimately, a major goal of experimental studies should be to inform THMC models for improved prediction of carbon mineralization at the field scale.

### 3.4 Coupled Fluid Flow and Mechanics for Stimulation

The existence or creation of a complex fracture network is needed to unlock the full carbon sequestration potential of subsurface mafic and ultramafic formations, especially considering the relatively low porosity and permeability of the rock matrix. Natural fractures are ubiquitous in the subsurface, but most of them are cemented or mineralized due to geological diagenesis at subsurface temperature and pressure conditions, suggesting low apparent permeability (Fu et al., 2021; Gale et al., 2014, 2018; Kostenko et al., 2002). As a result, methods of permeability enhancement, such as hydraulic fracturing and hydro‐shearing, may be needed to enhance the permeability of mafic and ultramafic rocks for sustained, extensive CO_2_ mineralization. Controlled laboratory experiments are essential to understanding permeability enhancement approaches because they allow for the identification of key mechanisms and the evaluation of optimization strategies. Here, we summarize key lessons learned from the permeability enhancement approaches used in the unconventional oil and gas revolution and enhanced geothermal system (EGS) development. We then identify the important knowledge gaps and future research topics to adapt the technologies for carbon mineralization in subsurface mafic and ultramafic rocks.

Extensive laboratory studies of hydraulic fracturing have been conducted on sedimentary, metamorphic, and igneous rocks, including shale (Tan et al., 2017), sandstone (Zoback et al., 1977), carbonate (Hou et al., 2018), phyllite (Oldenburg et al., 2020), schist (Farkas et al., 2019), gneiss (Boese et al., 2022), granite (Zhuang & Zang, 2021), and gabbro (D. Liu & Lecampion, 2022). The key experimentally identified parameters that affect hydraulic fracture initiation and propagation include rock microstructures, fluid viscosity, injection rate, stress conditions, and rock matrix permeability. Theoretical work also demonstrates several key physical processes strongly influencing hydraulic fracturing, such as borehole pressure diffusion, viscous fluid flow, creation of new solid surfaces, and leak‐off of fracturing fluid into porous fracture walls (Bunger et al., 2005; Detournay, 2016; Haimson & Fairhurst, 1967). These findings are mainly developed based on the coupling between fluid flow and rock deformation/fracturing. However, hydraulic fracturing of mafic and ultramafic rocks may involve additional physics—thermal effects and solid volume expansion—largely because certain minerals, such as olivine, will undergo hydration reactions upon contact with water‐based fracturing fluids that form volume‐expansion product minerals and generate significant heat (McCollom & Bach, 2009; Scott et al., 2017). Future studies are needed to explore the impact of thermal effect and solid volume expansion on hydraulic fracturing of mafic and ultramafic rocks.

There is an increasing interest in managing the complexity of hydraulic fractures in hydrocarbon‐bearing formations for enhanced oil and gas recovery, which can also benefit carbon mineralization in mafic and ultramafic rocks because a complex hydraulic fracture network will provide more reactive surface area for carbonation than simple fracture structures. Multiple hypotheses have been proposed to predict and manage hydraulic fracture complexity in hydrocarbon‐bearing formations. One popular hypothesis, pioneered by Renshaw and Pollard (1995), suggests that hydraulic fractures activate pre‐existing natural fractures, leading to a fracture network under weak horizontal stress anisotropy (Weng et al., 2011). Other studies suggest that the damage‐dependent Biot coefficient and its tensorial anisotropy can either promote or suppress the localization of multiple hydraulic fractures (W. Li et al., 2022; Rahimi‐Aghdam et al., 2019). The Biot coefficient governs the change of stress in the solid phase due to a change in fluid pressure with no change in the overall strain. Another recent experimental study indicates that the so‐called T‐stress, that is, the parallel stress near the crack tip, can cause fracture path curvature and strong localized stress concentration, eventually leading to fracture branching in homogeneous analog rocks (W. Li et al., 2022). Injection rate, fluid viscosity, and fluid chemistry have also been observed to strongly affect hydraulic fracture complexity (Barati & Liang, 2014; Ishida et al., 2004; W. Li et al., 2021). Future experimental studies are needed to investigate the mechanisms controlling hydraulic fracture complexity in mafic and ultramafic rocks, particularly accounting for the unique hydration and exothermic (heat releasing) reactions when water is in contact with olivine—one major constituent mineral in these rocks.

Hydro‐shearing is another means for permeability enhancement, often in EGS development. Hydro‐shearing involves the injection of fluid at pressures below or close to the minimum principal stresses in the reservoir to induce the shear slip of pre‐existing fractures. Shear slip of the pre‐existing fractures can cause self‐propping, hence permeability enhancement, due to the rough surfaces of the fracture walls. Numerous laboratory hydro‐shearing studies have been conducted on shale, granite, schist, amphibolite, and rhyolite, suggesting that in‐situ natural fractures must be permeable, weak, and favorably oriented to be hydro‐sheared (French et al., 2016; Kc & Ghazanfari, 2021; Meng et al., 2022; W. Wu et al., 2017; Ye & Ghassemi, 2018). After shearing, permeability enhancement differs significantly among different rock types, and in some cases, shear slip even causes decreasing fracture permeability (Z. Lei et al., 2023; Meng et al., 2022). It is currently unknown whether these findings apply to hydro‐shearing of fractures in mafic and ultramafic rocks, mainly because complex hydration, carbonation, and exothermic reactions can simultaneously occur during and after hydro‐shearing in these rocks. Laboratory experiments are needed to investigate these open questions because hydro‐shearing could provide a potential mechanism for enhancing CO_2_ storage potential in low‐permeability reservoirs during large‐scale CO_2_ mineralization operations.

Laboratory studies of hydraulic fracturing and hydro‐shearing on mafic and ultramafic rocks are crucial to identifying the key mechanisms for permeability enhancement to provide large reactive surface areas for CO_2_ mineralization. Furthermore, data collected from the controlled laboratory experiments will facilitate the development and validation of numerical models, with the ultimate goal of effectively predicting carbon mineralization at the field scale. However, it must be emphasized that hydraulic fracturing and hydro‐shearing, even when generating complex fracture networks, are expected to only provide initial surface areas for carbonation reactions. Sustained CO_2_ mineralization at scales will require significant fluid access to the rock matrix, which calls for an improved understanding of reaction‐driven damage/cracking, as discussed in the next section.

### 3.5 Coupled Flow‐Reaction‐Mechanics of Mineralization (HMC)

For low porosity and low permeability mafic and ultramafic rocks, CO_2_ storage volumes and reactive surface areas are limited to the pre‐existing fracture network. Thus, promoting storage in the long term requires processes that continually expand this network. Here, we review three coupled geochemical and geomechanical processes that could play a role in the evolution of fractures and reservoir reactivity during CO_2_ injection: (a) Reaction‐driven fracturing; (b) Sub‐critical fracture propagation; and (c) Enhanced reactivity of fracture systems.

#### 3.5.1 Reaction‐Driven Fracturing

The hydration of olivine and the carbonation of silicate minerals in mafic and ultramafic rocks results in a significant increase in volume, with the corresponding crystallization pressure estimated to be sufficiently large to fracture mafic and ultramafic rocks in the subsurface (e.g., Van Noort et al., 2017). For example, the reaction of olivine to magnesite and quartz yields a solid volume increase of 80%. However, the theoretical estimates of the crystallization pressure are an upper bound of the actual pressure exerted by the growing crystal, as it is calculated under the conditions of thermodynamic equilibrium. Without knowledge of the kinetics of the different reactions, it is not possible to assess the time it will take to cause microcracking of the rock and which reaction, hydration or carbonation, is mainly responsible for reaction‐driven cracking (Correns, 1949). For differential volumetric changes to work, carbonates must precipitate non‐uniformly (Xing et al., 2018); for crystallization pressure to work, carbonates must precipitate in interstices (grain boundaries, crystal defects) and have access to sufficient supersaturated fluids to grow and eventually crack the rock aggregate. Relevant field evidence for reaction‐driven fracturing exists in the spheroidal weathering of granite (Fletcher et al., 2006), basalt, and the serpentinization of peridotite (Jamtveit et al., 2009; Renard, 2021). Extensive carbonation of the Oman peridotite has been proposed as possible evidence of carbonation‐driven fracturing (Beinlich et al., 2020; Kelemen et al., 2011, 2018). Abundant listvenite outcrops― peridotite rocks that have been completely carbonated, exhibit brecciated textures with hierarchical fracture networks that are reflective of coupled carbonate deposition and fracture propagation (Beinlich et al., 2020; Falk & Kelemen, 2015; Kelemen et al., 2011). However, Menzel et al. (2022) argue that, based on observation of the core, the creation of persistent fluid pathways is primarily a function of tectonic stress and fluid over‐pressure rather than reaction‐driven cracking.

**Figure 6:**
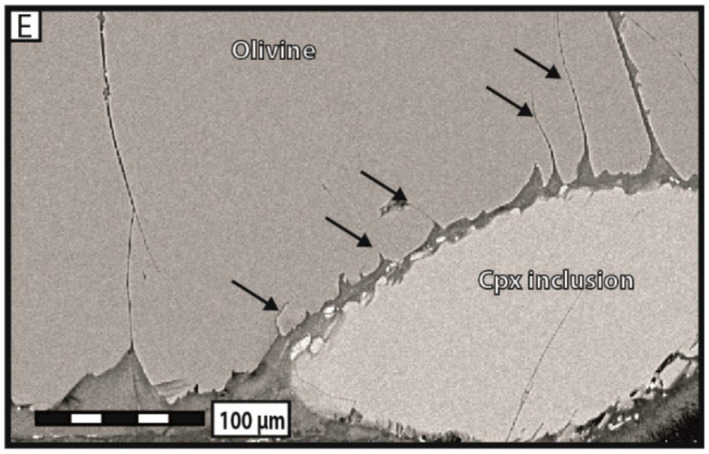
Volume‐increasing reactions under confining pressure could result in reaction‐driven fracturing during carbon mineralization operations. It is hypothesized that the induced fracturing could lead to increased permeability of the rock, and subsequently, a greater storage capacity. This figure shows experimental evidence of reaction‐driven fracturing of olivine by hydration and serpentine formation (Lafay et al., 2018).

Laboratory studies provide evidence of reaction‐driven fracturing during the evaporative precipitation of salt (Scherer, 2004), hydration of periclase (MgO) (Kuleci et al., 2017; Uno et al., 2022; Zheng et al., 2018), and the serpentinization of olivine, shown in Figure 6 (Lafay et al., 2018). However, experimental evidence for carbonation‐driven fracturing in mafic minerals is limited. While Lafay et al. (2018) observed clear evidence for hydration‐induced fracture propagation in olivine, they found no evidence for carbonate‐driven fracturing, possibly because carbonate reaction products were “delocalized” (i.e., did not concentrate on defects). Rather than searching for individual fractures, Van Noort et al. (2017) assessed the volumetric expansion of olivine aggregates exposed to high‐pressure CO_2_ and found limited evidence (only 1 of 17 triaxial experiments) for expansion, which they suggested was the result of precipitation clogging transport pathways, limiting continued reaction. W. Zhu et al. (2016) observed an increase in porosity in experiments on sintered olivine cups possibly due to reaction‐driven fracturing, but the deformation of the complex cup geometry created ambiguity in the interpretation. Xing et al. (2018) extended the work of W. Zhu et al. (2016) and argued that they observed reaction‐driven fracturing generated by non‐uniform distribution of precipitation and dissolution.

Whether or not reaction‐induced fracturing can occur depends on pore‐scale processes, including where carbonate crystals precipitate (on surfaces, in the solution, or focused on defects). The relative rates of growth and access to reactants are critical (Kelemen et al., 2019), as is the role of passivation and permeability reduction or enhancement. Different carbonation experiments have resulted in both the reduction in permeability of olivine‐rich peridotite (Lisabeth et al., 2017) and the increase in permeability at higher CO_2_ pressures (Andreani et al., 2009). It is unknown whether reaction‐driven processes occur in mafic rock such as basalt. Further investigation into reaction‐driven fracturing is needed to establish whether this process will play an important role during large‐scale industrial CO_2_ storage in mafic and ultramafic reservoirs.

#### 3.5.2 Subcritical Fracture Propagation

Chemical reactions have the potential to modify fracture toughness or the ease of fracture propagation (Atkinson, 1984; Eppes & Keanini, 2017). Because the subsurface is in many places “critically stressed” (Townend & Zoback, 2000), chemical reactions that weaken existing fractures can lead to spontaneous fracture initiation and propagation, called subcritical cracking (Brantut et al., 2013; Laubach et al., 2019). Although subcritical cracking is a localized failure, its occurrence will provide new fluid flow conduits for access to additional rock volumes, which may benefit the volumetric process of reaction‐driven cracking. There has been relatively little work conducted on the impacts of CO_2_‐induced reactions on rock strength and fracture propagation (Vafaie et al., 2023). Most of the existing work has been done on sandstone, showing variable but generally weakening impacts on rock strength, creep, and fracture behavior (Otu et al., 2023; Rinehart et al., 2016; Z. Wu et al., 2020; G. Zhang et al., 2020). An experiment on olivine‐rich peridotite (Lisabeth et al., 2017) found reduced strength, which they suggest may have involved sub‐critical fracturing during triaxial loading as a possible experimental demonstration of this behavior. However, much more work is needed on basalt and peridotite reactions with CO_2_ to assess the potential of subcritical fracture propagation to enhance carbonation.

#### 3.5.3 Enhanced Reactivity of Fracture Systems and Fracture Branching

**Figure 7:**
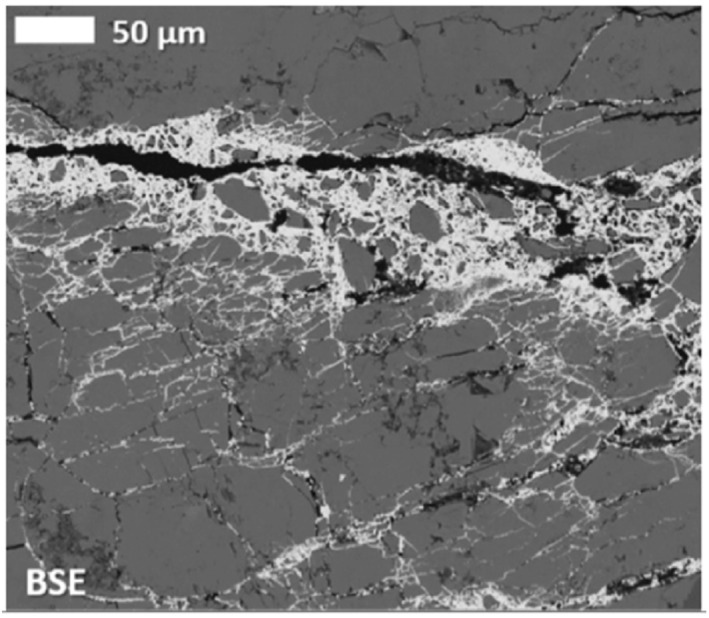
Fractured rock is expected to be more reactive with CO_2_ due to the generation of fine‐grained rock material, called fracture gouge, and the exposure of fresh mineral surfaces. This higher reactivity is demonstrated in this backscattered electron image of an experimentally shear‐fractured carbonate‐rich shale. The injected barium chloride solution resulted in the rapid (1 hr) precipitation of barium carbonate (bright‐white material; Menefee et al. (2020)).

Fracturing rock generates fresh mineral surfaces and comminuted material called fracture gouge (Reches & Dewers, 2005), which are likely to be more reactive with CO_2_‐bearing fluids. The gouge can include very fine‐grained and potentially strained or damaged material that has a greater reactive surface area (Tenthorey & Cox, 2006; Vrolijk & van der Pluijm, 1999). Additionally, newly‐exposed mineral surfaces lack thin, passivating layers (e.g., formation of nano‐scale hydration products) that can slow chemical reactions (e.g., Eisenlohr et al., 1999; Olsson et al., 2012). This higher reactivity has been demonstrated experimentally by Menefee et al. (2020), who provided evidence of enhanced carbonation within a fractured shale under dynamic fracturing experiments, in which a reactive solution was injected into newly created shear fractures (Figure 7). It is thus suspected that the natural or engineered activation of fractures in impermeable basalt or peridotite could be a possible mechanism to expose freshly formed and pre‐existing fractures to reactive fluids, maximizing the rate and amount of CO_2_ that can be stored. In addition, changes in stress induced by injection may reactivate existing shear fractures, producing more fracture gouge.

### 3.6 Challenges: Realistic Systems

One of the greatest challenges in predicting the long‐term viability of carbon mineralization in the field is connecting experimental studies to field observations. In this section, we review the mismatches between experimental and field results and the potential for reconciliation between the two. We focus on three potential areas of mismatch: (a) time‐ and length‐scale of reaction, (b) reactive surface area, and (c) differences in reaction conditions.

Regarding the time‐ and length‐scale of reaction, laboratory experiments are understandably conducted over limited time periods and spatial areas compared with field operations. Many reported experimental studies occur over weeks (Ennis‐King & Paterson, 2007; Raza et al., 2023; Romanov et al., 2015) to months (Gysi & Stefánsson, 2012; McGrail, Schaef, et al., 2009; McGrail, Sullivan, et al., 2009; Raza et al., 2023; Shibuya et al., 2013; Voigt et al., 2021), challenging their applicability to field sites. Even in the case of the rapid mineralization recently observed at CarbFix (Pogge von Strandmann et al., 2019) and Wallula (S. K. White et al., 2020), differences in the time scale of comparable experiments can result in overlooking mechanisms critical to predicting the long‐term viability of carbon storage, such as the passivation of reactive mineral surfaces (Kelemen et al., 2019). There are also challenges associated with scaling up pore‐ to column‐scale laboratory results. Mixing behavior is known to differ between the column‐ and field‐scale (Schincariol & Schwartz, 1990) and the field‐scale is likely to have much more spatial variability in reactive mineral facies (Deng et al., 2010).

Another source of mismatch between field and laboratory observations is the size of mineral reactants used in experiments. While increasing the reactive surface area through the use of fine‐grained powders can increase carbon sequestration per unit mass (Xiong, Wells, & Giammar, 2017), this is not representative of the nature of reacting surfaces during in situ geologic storage, which likely exists as consolidated rock (L. Li et al., 2008; M. Liu et al., 2022). Additionally, the use of powder samples cannot capture preferential precipitation on different mineral crystal faces, which has been observed for olivine (Olsson et al., 2012).

Finally, replicating the conditions of the subsurface field tests remains a challenge in the laboratory, due to the relatively slow kinetics of carbon mineralization of mafic and ultramafic rocks on a laboratory time scale. Therefore, elevated temperatures and solution compositions unrealistic of subsurface conditions in the field are frequently used in experiments to increase the rate of reaction, including temperatures exceeding 150°C (Z.‐Y. Chen et al., 2006; Garcia et al., 2010; Miller et al., 2019) and high concentrations of sodium bicarbonate (Z.‐Y. Chen et al., 2006; Gadikota et al., 2014; F. Wang et al., 2019). While these studies offer valuable insight into the mineralization process, the extreme conditions could potentially impact the saturation index with respect to carbonate mineral precipitation, and can therefore further impact the size (Q. Li et al., 2014), polymorphism (De Choudens‐Sánchez & Gonzalez, 2009), and location (e.g., homogeneous vs. heterogeneous precipitation; Amor et al., 2004) of secondary mineral precipitates.

Moving forward, a multi‐pronged approach is likely needed to advance our scientific knowledge in this area. While it remains necessary to tune experimental conditions to achieve reaction on a timescale observable in a laboratory setting, complementary experiments at natural conditions are necessary to distinguish differences in the reaction mechanism. Another promising approach is to conduct experiments using rock‐analog systems that can facilitate reactions while still capturing key aspects of the mineralization process (Osselin et al., 2016). These experimental insights are critical in identifying first‐order processes needed for developing realistic field‐scale simulations that can make actionable predictions of mineralization and capture the complex, coupled geochemical–geomechanical processes occurring in the subsurface.

## 4 Modeling and Simulation Studies Related to Carbon Mineralization

### 4.1 Introduction

The use and development of mathematical models and computational tools, guided by field and experimental data, is a necessary practical step if CO_2_ mineralization is to be predicted and realized at the field scale. Given our currently available computational tools, it is relatively infeasible to accurately predict the amount of CO_2_ mineralized as a function of time within a complex fractured system. Therefore, this section is dedicated to noting what models are available to initiate studies on CO_2_ mineralization in mafic and ultramafic rock. In addition, we describe the knowledge gaps of current modeling techniques and propose methods to fill these gaps.

CO_2_ mineralization within mafic and ultramafic rocks includes coupled THMC processes, which are poorly understood and not fully represented by current models. Temperature (T) has a critical effect on chemical reaction rates and mechanical properties. Hydrologic processes (H) control the transport of CO_2_ and chemical species necessary to dissolve rock and precipitate carbonates. Mechanics (M) determines the growth and closure of fractures that provide pathways for fluid to interact with fresh rock. Finally, chemical reactions (C) govern the kinetics of carbon mineralization in the system.

Experiments alone cannot provide sufficient information for models because key THMC parameter values require scale‐appropriate benchmark data to develop and validate the coupled models. Thus, there needs to be an integration of experiments and field data (both short‐term injections like CarbFix and natural analogs) to develop and validate models of the coupled processes involved in fracturing, injecting, and mineralizing CO_2_ in mafic and ultramafic rocks. We note that although models of some physical processes in the uncoupled endmembers of THMC are reasonably complete (e.g., flow in fractures, dissolution of minerals, strength of rocks), there are important processes that aren't adequately captured, such as passivation, sub‐critical cracking, reaction‐driven fracturing, and precipitation in fracture networks.

In many cases, separate thermo‐hydro‐mechanical (THM) and thermo‐hydro‐chemical (THC) models can be used to gain an understanding of mineralization in mafic and ultramafic rocks. THM models can help elucidate how the fracture network is created and THC models, including reactive transport models, can be used to predict how dissolution and precipitation affect flow and transport in the fractured system (Viswanathan et al., 2022). Combining these model types through the development of coupled thermo‐hydro‐mechanical‐chemical models is necessary for cases when there is strong feedback between mechanical and chemical processes. A major challenge will be to simulate THMC processes in complex fracture networks since THM and THC often operate at disparate time and length scales.

To move toward this objective, reduced complexity modeling (RCM) (Section 4.2) and Uncertainty Quantification (UQ) (Section 4.3) will be imperative first steps. RCM, which is based on models with first‐order couplings in simplified domains, can provide baseline insights into carbon mineralization processes and phenomena. This systematic approach to increasing complexity provides an initial step to coupling THMC phenomena in a single model. On the other hand, UQ can be implemented to account for poorly constrained input parameters in models, including inaccurate thermodynamic data, reaction rates, fracture parameters (e.g., fracture density, orientation, connectivity, etc.), and estimates of reactive surface area that may lead to grossly inflated mineralization rates in modeling studies. Once the key processes and uncertainties are defined, existing THM (Section 4.4) and THC (Section 4.5) models can be used for more detailed analyses of carbon mineralization. To arrive at a fully coupled THMC model of CO_2_ mineralization (Section 4.6), constrained by field and laboratory observations, it is imperative to overcome the key challenges outlined in Section 4.7.

### 4.2 Reduced Complexity Models

To unravel and understand the inner workings and couplings of key carbon mineralization phenomena, reduced complexity models can provide critical insight. Examples of how these models can be applied include: (a) classic scaling analysis (Barenblatt, 1996) focused on determining appropriate definitions of governing dimensionless groups (e.g., the Damköhler number) that control the transport and reaction of CO_2_‐charged fluids in the sub‐surface and (b) the development of models that capture and couple the first‐order phenomena in carbon mineralization. These efforts should incorporate models of transport, geomechanics, and geochemistry in one‐dimensional domains that consider the competing roles of phenomena such as dissolution and precipitation reactions, the impacts of dissolution‐ and precipitation‐induced porosity modifications on fluid flow, and the physical components of reaction‐driven fracturing. Ultimately, RCM models could build our understanding of key carbon mineralization phenomena and their interaction, identify the underlying time and space scales of carbon mineralization processes, prioritize the order of carbon mineralization phenomena, guide the effective design of laboratory experiments to provide key parameters for larger‐scale models, and determine which processes must be considered in the physics‐based models described in the forthcoming sections.

#### 4.2.1 Examples of RCMs to Provide Insight on THMC Processes

RCMs have been used in several efforts to better understand carbon mineralization. A recent study by Ratouis et al. (2022) was conducted to investigate CO_2_ mineralization during the CarbFix2 project in Iceland. The model considered non‐reactive solute transport and thermal effects while using a dual‐porosity type of model to represent the fractured basaltic reservoir, which covers an area of 42 km^2^. The tracer data measured to monitor the movement of the injected CO_2_ in the reservoir and the thermal data collected from the monitoring wells in the field were in agreement with the results of the numerical model, suggesting that, in certain cases, a non‐reactive simulation scheme can be used to monitor in‐situ mineralization, CO_2_ containment, and long‐term storage security in the reservoir. In addition, complementary geochemistry studies (Clark et al., 2020) showed that the processes of carbon and sulfur mineralization occurred fairly rapidly in CarbFix and CarbFix2 over timescales of months to years. The Carbfix and CarbFix2 pilot tests indicate that the permeability of the basaltic reservoir was barely affected by mineralization reactions over multiple years (Clark et al., 2020). This was attributed to the overall low volume percentage of mineral precipitation in the reservoir and the injected acidic fluids' tendency to dissolve the host rock close to injection well while precipitating minerals further away (Clark et al., 2020). The observation of mineral dissolution and thermally induced fracture growth near the borehole argues that coupling of mechanical deformation with reactive transport in a highly fractured basaltic reservoir is relatively weak. This is likely not the case in low‐permeability rocks such as peridotite, where permeability is expected to be affected by mineralization.

Evans et al. (2020) considered reaction‐driven cracking during the serpentinization of olivine with a model that combined reaction, deformation, and fluid flow. This model accounts for the presence and growth of cracks, which are described by a phase‐field model. Although phase‐field models are computationally expensive, they are used in this study to determine first‐order processes controlling reaction‐driven cracking. The model simulates the effect of the competition between cracking and porosity clogging on fluid pathway evolution. Although the study does not rigorously simulate coupled reactions, it demonstrates the positive feedback between an effective precipitation reaction and cracking, which leads to a self‐sustaining process. Notably, after introducing water diffusion due to chemical potential gradient into the numerical model, a crack pattern consisting of a primary path intersected by an orthogonal secondary set was reproduced, as has been observed in nature. Such a pattern resembles the so‐called “Frankenstein” crack frequently observed in peridotite outcrops in Oman (Evans et al., 2020).

### 4.3 UQ for Carbon Mineralization Applications

Uncertainty quantification is another critical tool needed to characterize CO_2_ mineralization, as both fracture and reaction parameters are difficult to constrain. Even if a fully coupled THMC model for CO_2_ mineralization in fractured mafic and ultramafic rocks were to exist, it could not be used to definitively predict the quantity of interest (QOI), that is, the temporal and spatial evolution of CO_2_ mineralization in mafic and ultramafic rocks. Rather, the model could be used to prescribe bounds on the QOI. This is due to the highly heterogeneous and uncertain nature of the fractured systems and the processes driving mineralization.

When considering engineering actions that could significantly increase CO_2_ mineralization rates, the following mechanisms are proposed: (a) Maximizing the reaction kinetics by dissolving CO_2_ in water (Sigfusson et al., 2015; Snæbjörnsdóttir et al., 2020) and adjusting pressure and temperature (Kelemen & Matter, 2008); (b) Increasing the rock surface available for reaction by pumping (injecting) the CO_2_ mixture through the fracture networks—natural or engineered—in the subsurface rock mass; (c) Creating self‐sustaining, reaction‐driven fracturing pathways by which the CO_2_ mixture can progressively advance from the fracture network into and through the bulk of the rock mass; and (d) Monitoring mineralization progress via remote sensing, for example, seismic imaging so that operational parameters can be adjusted as a function of time to maximize mineralization. The major caveat is that many of these processes are not well understood or characterized for mafic and ultramafic rocks. Key knowledge gaps revolve around a lack of a fundamental understanding of fluid flow and reactive transport in a fractured multi‐scale system, where the permeability, porosity, pore space geometry, and available surface area are continually changing through time. The primary uncertainties to consider are processes and parameters. The first uncertainty focuses on delineating the most important processes that influence the bounds of the QOI and the uncertainties in representing those with mechanistic models. The second uncertainty is centered on the parameters that play a role in the first‐order processes. These two aspects cannot be decoupled, since the uncertainty in model and parameter space has a significant influence on determining the most sensitive processes.

Identifying which processes have the most significant effects on carbon mineralization can be difficult because it is rare that one can observe the effects of a single process at the field scale. Often there are complementary or competing processes that make it impossible to isolate and quantify the impacts of a given process. Previous work has focused on the coupled effects of reactions and flow on the fate of solute transport through varying parameters such as the Damköhler number (Severino et al., 2012). As the mineralization of CO_2_ will depend upon the rates of precipitation and dissolution reactions as a function of the flow field, these parameters offer valuable insights into the behavior of dead‐end fractures, flow channeling, and other relevant phenomena. However, reaction rate constants utilized in these models are typically measured through batch laboratory experiments, which have shown significant variation compared to those observed in the field (Viswanathan et al., 1998). Attempts have been made to quantify the effects of such variations in key QOI such as solute‐to‐precipitate ratios (Srinivasan et al., 2007), where smaller laboratory‐scale experiments can provide invaluable information to make direct comparisons. For example, controlled laboratory experiments at reservoir temperatures, pressures, and stresses can mimic subsurface conditions to guide the optimization of mineralization at reservoir conditions. Experiments can also be designed to establish how to maximize reaction kinetics and create positive feedbacks to stimulate fracture branching and growth to access larger volumes of the reservoir rock. There will always be issues of scaling when applying models and parameters identified at the laboratory scale to field‐scale operations, however, significant insight can be gained from first‐order effects and associated uncertainties by this approach.

Recently, the use of Machine Learning (ML) algorithms in UQ has gained a lot of traction since these algorithms offer the ability to explore large dimensional models and parameter spaces very efficiently through the development of surrogate models. ML algorithms can also be used to query the sensitivity of processes relative to one another. Random Forest algorithms are perhaps one of the simplest ML techniques that have proven very powerful in their ability to identify the relative importance of models and parameters in various fields including geosciences (M. Liu et al., 2022; Srinivasan et al., 2018). As an example, a Random Forest algorithm can be used to infer whether rates for precipitation/dissolution reactions affect mineralization more than hydrogeological parameters like flow rate or fracture aperture, and how the topology or the fracture connectivity affects the amount mineralized.

Another popular use of ML algorithms is their ability, once trained, to accurately mimic complex multi‐physics problems at a fraction of the speed that it would take to run the original physics‐based models. As previously mentioned, mechanistic models of THMC processes in complex fracture networks would take several hours on High‐Performance computers, even when run in parallel. Since UQ requires several thousands, if not millions of runs to bound uncertainties in processes and parameters, this task becomes prohibitively expensive. For this reason, multi‐fidelity Monte Carlo models (Ng & Willcox, 2014; O’Malley et al., 2018; Peherstorfer et al., 2016) have been used very successfully to optimize available computational resources by utilizing a small number of high‐fidelity runs for learning the systematic biases between pairs of high and lower‐fidelity models.

Uncertainty quantification techniques can be used to characterize the relative uncertainties in reaction parameters and fracture/host rock properties and their impact on key quantities of interest such as carbon mineralized as a function of time. Parameters we have identified as uncertain include reactive surface area, reaction kinetics, thermodynamic data, fracture connectivity, fracture permeability, and matrix permeability. Therefore, we recommend that UQ techniques permeate all facets of carbon mineralization research including field observations, laboratory experiments, and computer simulation.

### 4.4 Thermo‐Hydro‐Mechanical (THM) Modeling of Fractures

Thermo‐hydro‐mechanical (THM) models have been used extensively to explore the physical processes involved in complex subsurface systems, including geothermal systems and unconventional reservoirs, at the pore and reservoir scale. Carbon mineralization has many commonalities to these systems with the exception that chemical reactions play an even more important role. Nevertheless, THM models can provide important information for understanding the structure of the fracture network and mechanical changes in fractures induced by fluid injection. THM models are generally categorized as discrete or continuum. In discrete models, the fracture is explicitly represented in the model. As a result, these models have been developed to simulate and predict complex fracturing processes such as fracture growth and branching. Continuum models use effective properties to consider existing fractures and do not consider fracture growth, but can model larger and more complex systems. Both continuum and discrete models have been applied at the pore and reservoir scale and will be discussed here. Given the wealth of existing studies and reviews on both thermo‐mechanical (TM; e.g., English, 2012; Ghassemi, 2012) and hydro‐mechanical (HM; e.g., Q. Lei et al., 2017) models and effects in fractured systems, we will focus our attention solely on thermo‐hydro‐mechanical (THM) models in this section, which are relatively few compared to TM, HM, and thermo‐hydro‐chemical (THC) models, the latter of which are discussed in the following section.

#### 4.4.1 Pore‐Scale THM Modeling

**Figure 8:**
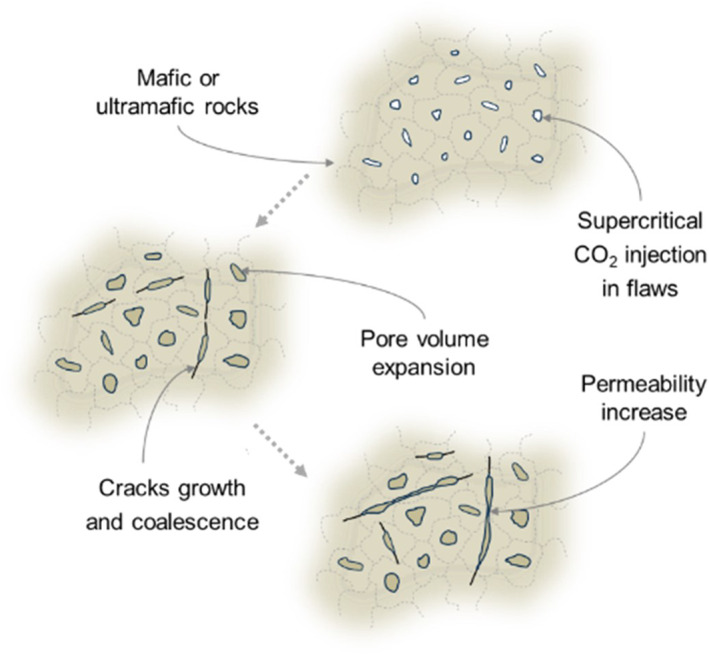
Schematic of crucial micro‐scale mechanisms responsible for the change of rock volume and fracture connectivity during carbon mineralization that require the formulation of multiscale chemo‐mechanical constitutive laws to be deployed within coupled HCM frameworks.

Carbon mineralization in mafic and ultramafic rocks involves important pore‐scale mechanisms that can alter the permeability, mechanical properties, and storage volume of the reservoir. Crucial micromechanical processes during CO_2_ injection include the interplay between reaction‐induced volume change, distributed microcracking, crack coalescence, and, ultimately, changes in bulk and/or fracture permeability (Figure 8). A promising avenue to accommodate these interactions is the incorporation of micromechanics theories into chemo‐poro‐mechanics constitutive frameworks. These efforts can benefit from analytical results by linking bulk mechanical properties to the microstructure of a solid matrix, such as the classic upper bound proposed by Hashin and Shtrikman (1963). Initial results in this area have recently been obtained in the context of micro‐poromechanics for water‐saturated geomaterials (e.g., Dormieux, 2006; Dormieux & Kondo, 2016; Kachanov & Sevostianov, 2018; Zaoui, 2002), providing a favorable avenue for future models focused on carbon mineralization.

Various other pore‐scale models that have been developed to simulate coupled THM processes include Discrete Element Method Pore Network Model (DEM‐PNM) (Al‐Busaidi et al., 2005; Shimizu et al., 2011), Discrete Element Method Computational Fluid Dynamics (DEM‐CFD) (Xiao‐Dong et al., 2015), and Lattice Boltzmann Method Discrete Element Method (LBM‐DEM) (Boutt et al., 2011; Z. Chen, Jin, & Wang, 2018; Z. Chen & Wang, 2017). In these models, mechanical interactions are solved by the discrete element method while the fluid flow equation is solved by either a pore network model, computational fluid dynamics, or lattice Boltzmann method. All three models have been applied to simulate hydraulic fracturing and achieved some success. Particularly, the coupled LBM‐DEM model has been used to study the effect of strength heterogeneity on fracturing patterns and microfailure mechanisms (Z. Chen & Wang, 2017). It has also been used to investigate hydraulic fracture propagation in rocks with cemented natural fractures (Z. Chen, Yang, & Wang, 2018). The numerical results show that both the strength ratio (between cemented natural fractures and the host rock) and the approach angle (between hydraulic and cemented natural fractures) significantly affect hydraulic fracture propagation. These pore‐scale coupled hydro‐mechanical models have provided important insight into rock failure and fracture initiation and propagation mechanisms. These mechanisms may be important to consider during the injection of CO_2_ into low‐permeability rocks such as peridotite.

#### 4.4.2 Reservoir‐Scale THM Modeling

To predict CO_2_ mineralization in mafic and ultramafic rocks, it is necessary to consider THM processes and responses in fractured systems at the reservoir scale to changes in effective stresses, flow conditions, and temperatures as part of the modeling framework (Stephanson et al., 1997). For instance, fracture growth due to imposed hydro‐mechanical processes (e.g., hydraulic fracturing) would create additional surface area for CO_2_ mineralization, whereas fracture sealing due to lithostatic load/stress changes would decrease surface area. Combining the potential thermal effects, such as the geothermal gradient in the crust or temperature changes due to reactions, with such mechanical aspects would also need to be considered because of the known feedbacks between thermal and mechanical effects.

In general, reservoir scale THM models operate by simulating fracture growth due to stress and thermal effects (discrete) or use discrete fracture networks and impose an external stress field on a pre‐existing network of fractures to determine which fractures open and close as a function of time (continuum). A discrete mechanistic model for fracture growth is the combined finite‐discrete element model (FDEM). FDEM is an appealing THM model for mineralization since it can simulate fracture growth due to stress and fluid effects. Details of FDEM can be found in Munjiza (2004). FDEM can effectively simulate fracture processes because underlying mechanisms of crack initiation, propagation, and coalescence in geologic materials can all be captured (Knight et al., 2020). These fracture processes are critical for predicting geologic carbon storage since subcritical and reaction‐driven cracking may play an important role in accessing enough host rock for scalable carbon mineralization. Wide ranges of length and time scales can be simulated with FDEM‐based models, from sub‐mm to km, and ns to months, respectively. However, in practice, it is nearly impossible to use pure FDEM models for extended time period 3D simulations (>hours) due to time‐stepping issues, which are discussed below. For this reason, the mechanics of fracture growth, which tends to be a shorter time scale process, has historically been treated separately from chemical reaction, which is a long‐time scale process. This separation of time scales enables a simplification of the analysis by uncoupling the fracture propagation from the chemical reactions. However, in other problems such as reaction‐driven cracking, such simplification is not possible as it is the chemical processes that are driving the mechanical damage.

There are only a few examples of THM FDEM models in the literature and even fewer are capable of modeling 3D systems. Yan et al. (2022) developed a 2D THM FDEM model within the MultiFracs FDEM software that can simulate fluid and heat transport in complex fracture networks, as well as crack initiation, propagation, intersection, and closure due to THM effects. Sharafisafa et al. (2023) also developed a 2D THM FDEM model within the Irazu geomechanical software that can model both fracture creation/growth and pre‐existing discrete fractures. Another example is the Hybrid Optimization Software Suite (HOSS), which is a hybrid multi‐physics platform based on FDEM. HOSS allows for preexisting discrete fractures in the rock to be explicitly modeled and used in both solid and fluid domains. One of the limitations of the applications of FDEM models to carbon mineralization is the restrictions placed upon the maximum allowable time step size because a fracture aperture tends to be much smaller than the rock element sizes. The small length scales require small FDEM timesteps, limiting their applicability at the reservoir scale. That being said, FDEM models have been used recently to simulate fracture propagation and flow in fractures, both key processes for carbon mineralization at the meter to 10 m scale (Z. Lei et al., 2019).

Discrete fracture network (DFN) and discrete fracture matrix (DFM) models differ from the FDEM models previously described in the sense that they do not consider fracture growth or coalescence. Instead, they assume the majority of fractures already exist, and external stresses simply open and close these fractures. The advantage is that more complicated physics at larger scales can be more easily simulated, often using existing software. THM models coupled to DFNs are prevalent in the literature for a range of application spaces that involve subsurface fractured systems (Birkholzer et al., 2021; Cui & Wong, 2021; Garipov & Hui, 2019; Z. Lei et al., 2023; S. Li et al., 2019; Liao et al., 2023; Pandey et al., 2017; Stefansson et al., 2021; F. Wang et al., 2019; Yao et al., 2018). THM DFN models can also capture key feedback between aperture changes related to heat and changes in fluid pressure and/or in situ stress conditions. These processes are particularly important during the injection phase of an operation such as CO_2_ injection or geothermal energy production. An example of their application is described by Nadimi et al. (2020), who developed a 3‐D DFN model to simulate an optimum hydraulic fracture network for Phase 3 injection and production wells at the Frontier Observatory for Research in Geothermal Energy (FORGE) site in Utah. Using this method, the authors identified optimal operating conditions and principal parameters (injection temperature and flow rate) that govern production quality.

FDEM with a coupled fluid solver can aid in understanding the fracture network structure, geometry, and topology created by fluid‐driven cracks within the rock, with the key drawback that likely only short timescales can be modeled. On the other hand, continuum models, such as DFN and DFM models coupled to THM simulators, can capture more complex fracture networks but are not capable of generating new fractures, which could prove critical under certain mineralization conditions. Both of these coupled models can capture more complex fracture networks and can account for changing fracture permeabilities and porosities using Barton‐Bandis‐type analytical equations or fully coupled physics models. Results from these models can give one an estimate of the available surface area for mineralization and garner information needed to predict how much CO_2_ can be mineralized in a fractured system.

To overcome the limitations of each model, a possible solution is to employ both models and leverage the strengths of each. An excellent example of this is provided by Birkholzer et al. (2021), whose authors used this integrated modeling framework to predict fracture network evolution during the initiation of hydraulic fracturing at the Hydraulic Fracturing Field Test (HFTS) in the Permian Basin (USA). The authors coupled two high‐performance simulators; GEOS, a FDEM‐type geomechanical code designed to model hydromechanical variations during hydraulic fracturing initiation; and TOUGH+, a continuum code used for multi‐phase flow for production, to arrive at a reservoir‐scale model (Birkholzer et al., 2021). This example provides promising advancement in the application of THM models to complex reservoir‐scale processes. Since TOUGH+ also has reactive transport capabilities, geochemical modeling can also be conducted, making these models quite useful for carbon mineralization calculations. The main feature lacking in these models is a mechanistic representation of fracture growth, which could be important during the injection phase if hydraulic fracturing is creating new fractures or if reaction‐driven cracking is a dominant mechanism.

### 4.5 Thermo‐Hydro‐Chemical (THC) Modeling of Fractures

As fluids pass through fractures, which are the primary pathways through low‐permeability rock in the Earth's subsurface (Bonnet et al., 2001; Deng & Spycher, 2019; National Research Council, 1996; Neuman, 2005; The National Academies of Sciences, Engineering, and Medicine, 2021; Viswanathan et al., 2022), they are commonly out of equilibrium with the resident minerals and a variety of chemical reactions, for example, dissolution and precipitation, will occur (Deng & Spycher, 2019; Laubach et al., 2019). These reactions control carbon mineralization rates in mafic and ultramafic rock and can alter the passage of fluid by changing the permeability of the system. Incorporating these chemical reactions into simulations is, thus, an effective tool for identifying key parameters that can hinder or optimize mineralization rates.

In contrast to THM modeling, a large amount of literature exists on THC modeling (e.g., reactive transport modeling) that is relevant to carbon mineralization in fractured systems. This class of model is capable of estimating relevant mineralization timescales if the input parameters can be constrained and adequate benchmark data can be provided. There are a variety of THC simulator options that differ in terms of spatial dimensions, discretization schemes, time integration methods, governing equations, flow simulator capabilities (single‐phase Darcy flow, variable saturation Richards flow, multi‐phase flow, variable density, non‐isothermal, and heterogeneous permeability), transport formulations (advection, mechanical dispersion, molecular diffusion, multi‐continuum), and geochemistry options (surface complexation, kinetic mineral precipitation‐dissolution, aqueous kinetics, mineral nucleation, mineral solid‐solutions). The most common simulators used today are PHREEQC (Parkhurst & Appelo, 2013) which is the geochemistry engine for HPx (Jacques et al., 2018), PHT3D (Prommer et al., 2003), OpenGeoSys (Kolditz et al., 2012), HYTEC (van Der Lee et al., 2003), ORCHESTRA (Meeussen, 2003), TOUGHREACT (T. Xu et al., 2011), eSTOMP (M. D. White & Oostrom, 2003), HYDROGEOCHEM (Yeh & Tripathi, 1990), CrunchFlow (Steefel, 2009), MIN3P (Su et al., 2021), and PFLOTRAN (Lichtner et al., 2015). A comparison of strengths and weaknesses between the codes is provided by Steefel et al. (2015).

Recently, partially complete, coupled models that include thermo‐hydro‐chemical (THC) processes in fractured porous media have been used to simulate geochemical reactions during carbon sequestration (Dai et al., 2020 and references therein). In this section, we present an overview of currently available flow and reactive transport simulation codes in the context of subsurface porous and fractured media with a specific focus on their utility in modeling carbon mineralization at the pore and reservoir scale.

#### 4.5.1 Pore‐Scale THC Models

In recent decades, pore‐scale models have been increasingly developed and utilized due to rapid advancement in imaging techniques, which provide detailed pore structures for numerical simulations, and rapid advancement in supercomputer architectures, which enable pore‐scale simulation of rather large systems, sometimes allowing direct comparison between pore‐scale simulations and experiments (L. Chen et al., 2022). Pore‐scale models directly solve governing equations using fully resolved pore structures. Many studies have been focused on mineral dissolution in fractured porous media considering the resultant pore‐structure change (L. Chen, Kang, Carey, & Tao, 2014; L. Chen, Kang, Viswanathan, & Tao, 2014; Q. Kang et al., 2002, 2014; M. Liu & Mostaghimi, 2017; Rasoulzadeh et al., 2020; Starchenko et al., 2016; Verberg & Ladd, 2002; Verhaeghe et al., 2005, 2006). These studies identified different dissolution regimes (e.g., uniform dissolution, face dissolution, wormholing) based on the combination of Peclet and Damköhler numbers, as well as conditions for transition between different regimes (Q. Kang et al., 2014). Further investigation of coupled dissolution and precipitation in two‐dimensional (2D) domains has been carried out, where the effect of precipitation of a secondary solid phase on the dissolution of the primary solid phase is investigated under varying reaction kinetics, molar volume, surface roughness, and nucleation and crystal growth mechanisms (L. Chen, Kang, Carey, & Tao, 2014; L. Chen, Kang, Viswanathan, & Tao, 2014; Q. Kang et al., 2010). More recently, these models have evolved to 3D‐space, where dissolution and precipitation in 3D fractures was simulated with evolving fracture geometry (Q. Kang et al., 2023). These collective pore‐scale studies have improved our understanding of the complex coupling between fluid flow, solute transport, dissolution/precipitation, and evolution of pore structures and provided physics‐based constitutive parameters for large‐scale models of coupled THC processes.

#### 4.5.2 Reservoir‐Scale THC Models

THC reservoir‐scale models are highly relevant for studying carbon mineralization in fractured systems since they capture the interplay between dissolution, precipitation, and fluid flow at a representative scale. In contrast to the pore‐scale models described in the previous section, these models make continuum approximations to simulate larger scales. Specifically, continuum models do not explicitly consider features such as surface roughness in fractures but instead, use effective properties such as heterogeneous porosity and permeability to capture the effects of roughness. These models simulate key chemical reactions, such as dissolution and precipitation, by modifying the permeability and porosity within each computational cell. The modification of the porosity is used to update the rock's permeability using a constitutive relationship between permeability and porosity. The most common constitutive relation used in THC models is Kozeny/Kozeny‐Carmen‐like equations (Bear, 1988; Carman, 1939, 1956; Kozeny, 1927). However, further study is needed to determine if a more rigorous pore‐scale relationship between reaction and permeability/porosity warrants modification of Kozeny‐Carmen‐like equations to treat mineralization.

Characterizing the feedback between the network structure, flow field, and associated reactive transport during carbon mineralization requires a coupled THC simulator capable of dynamically modifying flow resistance (hydraulic aperture/permeability) within a three‐dimensional fracture network. The addition of fractures, however, adds a unique element of complexity to THC modeling. Within these models, fractures are mostly treated indirectly using effective parameters, where the fracture walls are not explicitly represented. Rather, most models retain a continuum formulation where the porosity and permeability of the cell capture the higher permeability of the fracture. To date, the inclusion of fractures into THC simulations has largely been carried out in a single fracture, small two‐dimensional networks, or in upscaled/equivalent continuum models (Andrews & Navarre‐Sitchler, 2021; Andrews et al., 2023; Deng, Molins, et al., 2018; Deng, Steefel, et al., 2018; Feng et al., 2019; Jones & Detwiler, 2019; Lebedeva & Brantley, 2017; Molins et al., 2019; Noiriel et al., 2021; Pandey & Rajaram, 2016; Steefel & Hu, 2022; Steefel & Lasaga, 1994; Steefel & Lichtner, 1998). Recently, however, THC modeling in 2D/3D DFN and DFM models has begun to take place, cf., Y. Tsang et al. (1996), Jackson et al. (2000), Lichtner (2000), Berkowitz (2002), MacQuarrie and Mayer (2005), Neuman (2005), Hartley and Joyce (2013), Hadgu et al. (2017), Steefel and MacQuarrie (2018), Iraola et al. (2019), Sweeney et al. (2020), Andrews and Navarre‐Sitchler (2021), Pachalieva et al. (2023), Andrews et al. (2023), and references therein.

**Figure 9:**
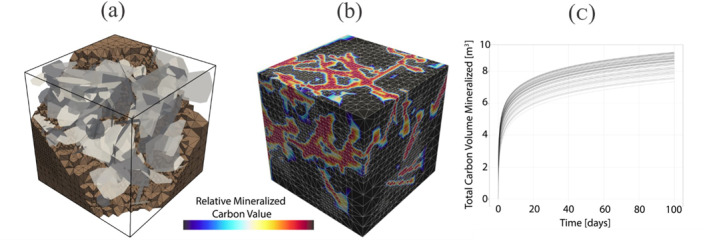
An example of a discrete fracture network inserted into a rock mass, allowing for a standard finite volume THC code to simulate mineralization reactions within heterogeneous flow fields. (a) A discrete fracture‐matrix representative fracture network in a mafic rock. Fractures are gray discs and the rock matrix is shown in brown. (b) Results of simulating dissolution and precipitation reactions in the fracture network and rock matrix. Here we see the spatial variability in the mineralized carbon due to the heterogeneity created by the inclusion of the fracture network. (c) Estimates of the volume of CO_2_ mineralized under different geochemical conditions with varying rock and fracture properties. These models allow researchers to test the relative importance of hydrological, geological, and geochemical properties on the total volume of carbon that can be mineralized.

Three‐dimensional high‐fidelity simulations, although heavily sought after, have been relatively infeasible due to computational limitations. However, recent developments in high‐performance computing now allow for the exploration of flow and reactive transport properties in 3‐D fractured media (Hyman, Navarre‐Sitchler, et al., 2022; Hyman, Sweeney, et al., 2022). Figure 9 shows the results of a reactive transport simulation in fractured media using the dfnWorks software suite (Hyman et al., 2015). The fractures have a permeability that is multiple orders of magnitude times higher than that of the surrounding matrix. As fluid out of equilibrium is injected into the domain, carbon is mineralized. Note that these simulations only capture thermo‐hydro‐chemical coupling, not mechanical changes. Figure 9a shows the fracture network and rock matrix, Figure 9b shows the spatial variability of mineralized carbon at the end of the simulation, and Figure 9c shows the total volume of mineralized carbon for different realizations of the network but under the same hydro‐chemical scenarios. These recently developed 3‐D simulations offer a promising technique for estimating the amount of CO_2_ that can be mineralized in a fracture network.

The most physically representative simulation tools for carbon mineralization systems are DFM methods where the matrix surrounding the fracture network is included in the model (Berre et al., 2019; Hyman, Navarre‐Sitchler, et al., 2022; Hyman, Sweeney, et al., 2022). Because CO_2_ could mineralize in both the fractures and rock matrix, the inclusion of both is warranted. Special numerical formulations, such as mimetic finite difference, are required for DFM models (Lipnikov et al., 2014). Chemistry is simulated using codes such as Alquimia, which allow for RTM to be performed (Andre et al., 2013; Hyman, Navarre‐Sitchler, et al., 2022; Hyman, Sweeney, et al., 2022). Fracture software such as dfnWorks (Hyman et al., 2015) can be used with RTM models such as PFLOTRAN, FEHM (Zyvoloski, 2007), and AMANZI (Moulton et al., 2012). The large number of THC models and fracture software allow for a comprehensive study of THC mineralization processes at the reservoir scale.

A challenge for simulating field scale mineralization is that the apparent mineral dissolution rate can be orders of magnitude lower than the laboratory‐measured rates (Andrews & Navarre‐Sitchler, 2021; Atchley et al., 2013; Beisman et al., 2015; Jung & Navarre‐Sitchler, 2018a; A. F. White & Brantley, 2003). In turn, many of these systems are transport‐controlled and are not solely a function of the kinetic parameters used in standard geochemical models. These slow apparent dissolution rates are more common in fracture networks than homogeneous porous media due to the heterogeneous flow field induced by the fracture network (Andrews & Navarre‐Sitchler, 2021; Andrews et al., 2023; Becker & Shapiro, 2000; Berkowitz & Scher, 1997; Edery et al., 2016; Geiger et al., 2010; Haggerty et al., 2001; Huseby et al., 2001; Hyman et al., 2019; Jung & Navarre‐Sitchler, 2018a, 2018b; P. Kang et al., 2020; Meigs & Beauheim, 2001; Painter et al., 2002; Pandey & Rajaram, 2016; Wen & Li, 2018). However, since these models do not incorporate mechanical effects, they cannot simulate coupled geochemical‐mechanical processes such as reaction‐driven cracking, subcritical cracking, or fracture reactivation/closure due to mineralization, which are likely critical for accessing sufficient host rock to make mineralization feasible. As with the THM models, pore‐scale processes need to be abstracted and included in reservoir models. This is more straightforward for THC models since the reactive models employed at each spatial location are often the same in pore and reservoir scale models.

### 4.6 Thermo‐Hydro‐Mechanical‐Chemical (THMC) Modeling

Developing a fully coupled THMC model is a challenging task due to the disparate timescales of THC and THM processes. Geochemical processes modify the fracture's hydraulic resistance (Ellis et al., 2013) and can drive or inhibit fracture propagation (Detwiler & Morris, 2018; Shovkun & Espinoza, 2019). For example, reaction‐driven cracking, which may be an important process during carbon mineralization in low permeability rocks such as peridotite, would need a model that couples the chemical effects of dissolution and precipitation with mechanical effects that lead to fracturing of the rock. Alternatively, chemical reactions in fractured rock may lead to subcritical cracking by weakening the rock, resulting in fracturing due to existing or induced stress. Evidently, to arrive at a comprehensive multi‐scale model that can capture critical carbon mineralization processes, THC and THM models need to be integrated.

To date, very few THMC studies related to carbon mineralization have been published. That being said, attempts have been made at the pore‐scale in areas such as serpentinization and retrograde metamorphism, and thus, similar efforts could be applied to carbon mineralization in mafic and ultramafic rocks. Although not a fully coupled model, M. Liu and Mostaghimi (2017) investigated the impact of dissolution on the mechanical properties of porous media through pore‐scale simulations. In their study, pore structures of sandstone and carbonate rocks before and after dissolution were used to predict their mechanical properties (effective Young's modulus and Poisson's ratio). Their simulation results showed a strong dependency of mechanical properties on the dissolution regime characterized by the Peclet and Damköhler numbers. Nonetheless, their simulations of HC and mechanical processes are not coupled. Malvoisin et al. (2017) used a pore‐scale fracture mechanics method to compute the serpentinization‐induced propagation of a Mode I fracture from an initial etch pit in an olivine grain. The authors observed an increase in reactive surface area due to micro‐cracking and found that the related evolution of bulk reaction rates compared well with experimental data on olivine powders. Other THMC‐relevant examples are found in Jamtveit et al. (2008), L. Zhang et al. (2019), and Y. Zhang et al. (2019). Jamtveit et al. (2008) used a 2D spring‐network model of a rock undergoing a retrograde metamorphic reaction. The model shows that the network of micro‐cracks resulting from the induced stress heterogeneity assists the migration of fluid in a rock that has a negligible initial permeability. It supports the hypothesis that the increase of solid volume accompanying retrograde metamorphic reaction is critical for ensuring a self‐sustaining process. L. Zhang et al. (2019) and Y. Zhang et al. (2019) utilized a 3D FDEM to analyze the fracturing induced by volumetric expansion during a metamorphic reaction. The model is capable of reproducing many features observed in the periclase hydration experiment reported by Zheng et al. (2018), such as the existence of a porosity pulse accompanying the moving reaction front.

At the reservoir scale, the feasibility of using scCO_2_ as a working fluid in geothermal reservoirs was assessed by Gan et al. (2021). The THMC model incorporated changes in mineral dissolution and precipitation during the injection of scCO_2_ and the resultant evolution of the permeability and porosity of the reservoir. The authors determined that injecting CO_2_ could be a favorable option as it increases the permeability and porosity of the aquifer more effectively than water‐based circulation and is more capable of extracting heat energy from the rock.

These studies show that some progress has been made on fully coupled THMC at the pore and reservoir scale. While the examples provided above are relevant, specific models that incorporate the key geochemical and mechanical processes that occur during carbon mineralization are necessary. To arrive at this, key challenges need to be addressed, which have been discussed above and are summarized below.

### 4.7 Challenges With the Development of Representative Carbon Mineralization Models

Several challenges exist with the development of realistic carbon mineralization models that incorporate all aspects of carbon mineralization. Current models either specialize in THM or THC processes and a fully coupled THMC model does not exist at the reservoir scale. Given that the development of a successful carbon mineralization operation is likely to depend on the optimization of the coupled effects between geochemistry and geomechanics to create positive feedback to access sufficient host rock, moving toward a fully coupled model is a priority. The disparate timescales of THM and THC make a fully coupled model problematic. Model decomposition techniques that correctly model short‐ and long‐term scale processes are a possibility. Pseudo‐steady state assumptions can also be attempted.

Carbon mineralization is inherently a multi‐scale problem and therefore large‐scale macroscopic field observations, controlled small‐scale laboratory experiments, and reduced complexity models are needed to inform and constrain multi‐scale simulations that include UQ. Another challenge is unconstrained, uncertain, and unknown reaction and fracture parameters, which could yield inaccurate results that are not reproducible at the field scale. Robust and representative experimental studies are needed to accurately derive these parameters.

## 5 Conclusions and Outlook

To mitigate the current global climate crisis, immediate action must be taken to reduce atmospheric CO_2_ levels. Carbon mineralization in mafic and ultramafic geologic reservoirs has been proven through recent field studies at CarbFix1 and CarbFix2 in Iceland and Wallula in Washington, USA, to be a promising means for quickly and securely storing CO_2_ in the subsurface. However, many unknowns remain that must be addressed before storage operations can be scaled up and expanded to other favorable subsurface lithologies. In this review, we have outlined the critical first‐order processes that must be considered while exploring this complex system, outstanding questions to be answered through experimental and modeling efforts, and ongoing pilot‐scale field tests.

First, regarding the field scale studies of carbon mineralization, while rapid and highly efficient mineralization was observed in CarbFix, CarbFix2, and Wallula, generalization of these results to other potential field sites necessitates answering key questions including (a) whether upscaling using CO_2_‐saturated water can be feasible given the water requirements or if supercritical CO_2_ injection is needed to scale‐up efforts; (b) whether performance can be maintained over extended periods; and (c) how these findings can be applied to less permeable lithologies. The last question is currently being explored through field tests in relatively impermeable Oman ophiolite, however, it opens the door to many further questions surrounding how mineralization within fractured systems will impact the connected fracture network. These include whether reaction‐driven fracturing and sub‐critical fracturing could enhance storage volumes and formation permeability, or whether carbonate precipitation leads to passivation of reactive surfaces or fracture clogging, quickly depleting the storage potential.

Second, we refine the extensive published literature on experimental studies of carbon mineralization to gain insight into how this reaction will proceed within fractured systems. While many studies exist to quantify the rates of carbonate mineral precipitation, particularly from the reaction of CO_2_ with olivine, major uncertainties remain as to how this reaction will occur within fractures in the field, where reaction rates will be a function of the available surface area and the extent of reaction front penetration into an impermeable rock is uncertain. Flow through laboratory experiments can better reflect the reaction system in the field, where the relative reaction versus transport rates will greatly influence mineralization regimes. However, these experiments must also capture the impacts of the reaction on fracture propagation and activation. Lastly, differences between experimental studies and field observations must be considered. This includes the effects of surface passivation and differences in reaction conditions, typically employed to accelerate reaction rates to be observable at faster timescales. The effects of field‐scale fracture networks and connectivity on these reaction processes must also be considered.

Finally, we explore currently available mathematical models and computational tools for simulating carbon mineralization on the pore‐to‐reservoir scale and make recommendations for improving these simulations to capture critical mechanisms observed through experiments and in the field. The biggest perceived challenge is coupling developed thermo‐hydro‐mechanical and thermo‐hydro‐chemical models to THMC models, which can simulate the simultaneous dissolution and precipitation processes that drive carbon mineralization while also capturing how this process can drive fracturing through stress corrosion and reaction‐driven fracturing. An equally important challenge is that many parameters needed for models must still be constrained through further experiments and field studies or the models will not be predictive. Defensible predictions within uncertainty bounds of field‐scale carbon mineralization in fractured systems, achieved through closing the knowledge gaps outlined in this review, will enable us to take full advantage of this strategy to reduce atmospheric CO_2_ emissions and address the current climate crisis.

## Conflict of Interest

The authors declare no conflicts of interest relevant to this study.

## Erratum

The originally published version of this article contained an error. In Figure 1, the labels for experimental and field scales were swapped. The error has been corrected, and this may be considered the authoritative version of record.

## Data Availability

Data were not used, nor created for this research.
